# Coordination tailoring of Cu single sites on C_3_N_4_ realizes selective CO_2_ hydrogenation at low temperature

**DOI:** 10.1038/s41467-021-26316-6

**Published:** 2021-10-15

**Authors:** Tang Yang, Xinnan Mao, Ying Zhang, Xiaoping Wu, Lu Wang, Mingyu Chu, Chih-Wen Pao, Shize Yang, Yong Xu, Xiaoqing Huang

**Affiliations:** 1 State Key Laboratory of Physical Chemistry of Solid Surfaces, College of Chemistry and Chemical Engineering, Xiamen University, Xiamen, China; 2 Institute of Functional Nano & Soft Materials (FUNSOM), Jiangsu Key Laboratory for Carbon-Based Functional Materials & Devices, Soochow University, Suzhou, China; 3 Guangzhou Key Laboratory of Low-Dimensional Materials and Energy Storage Devices, Collaborative Innovation Center of Advanced Energy Materials, School of Materials and Energy, Guangdong University of Technology, Guangzhou, China; 4 National Synchrotron Radiation Research Center, Hsinchu, Taiwan; 5 Eyring Materials Center, Arizona State University, Tempe, AZ USA

**Keywords:** Heterogeneous catalysis, Energy, Materials for energy and catalysis

## Abstract

CO_2_ hydrogenation has attracted great attention, yet the quest for highly-efficient catalysts is driven by the current disadvantages of poor activity, low selectivity, and ambiguous structure-performance relationship. We demonstrate here that C_3_N_4_-supported Cu single atom catalysts with tailored coordination structures, namely, Cu–N_4_ and Cu–N_3_, can serve as highly selective and active catalysts for CO_2_ hydrogenation at low temperature. The modulation of the coordination structure of Cu single atom is readily realized by simply altering the treatment parameters. Further investigations reveal that Cu–N_4_ favors CO_2_ hydrogenation to form CH_3_OH via the formate pathway, while Cu–N_3_ tends to catalyze CO_2_ hydrogenation to produce CO via the reverse water-gas-shift (RWGS) pathway. Significantly, the CH_3_OH productivity and selectivity reach 4.2 mmol g^–1^ h^–1^ and 95.5%, respectively, for Cu–N_4_ single atom catalyst. We anticipate this work will promote the fundamental researches on the structure-performance relationship of catalysts.

## Abstract (web summary)

CO_2_ hydrogenation has attracted intense scientific attention yet suffers from the disadvantage of poor activity and low selectivity. Here, the authors report that Cu single atom catalysts with tailored coordination environments on C_3_N_4_ serve as highly selective catalysts for CO_2_ hydrogenation.

## Introduction

CO_2_ hydrogenation has attracted great attention because it can simultaneously reduce the CO_2_ emission^1–4^ and produce high value-added chemicals^5–8^, yet it suffers from low activity and selectivity due to the chemical inertness of CO_2_ and the complicated process of CO_2_ hydrogenation^9–11^. To promote CO_2_ hydrogenation, one needs to raise the temperature or pressure, which however results in increased selectivity of CO and decreased selectivities of value-added chemicals. For instance, Cu-based catalysts have been widely used for CO_2_ hydrogenation, which are generally operated at temperature and pressure ranges of 220–300 °C and 50–100 atm, respectively^10,11^. Over the past decades, great efforts have been devoted to developing efficient catalysts for CO_2_ hydrogenation, such as compositing Cu sites with metal oxides (e.g., ZnO, ZrO_2_, and TiO_2_)^12–20^. However, the request for efficient CO_2_ hydrogenation catalysts is driven by the current drawbacks including poor activity and/or CH_3_OH selectivity, as well as the ambiguous mechanism.

Recently, single atom catalysts (SACs) have attracted great attention in catalysis due to their properties including 100% atom utilization efficiency, low coordination environment, confinement effect, etc^21–30^. Typical for CO_2_ hydrogenation, SACs are regarded as promising catalysts for producing high value-added chemicals. For instance, Ye et al. synthesized Ir_1_–In_2_O_3_ SAC for CO_2_ hydrogenation with a high C_2_H_5_OH selectivity of 99% and a turnover frequency (TOF) value of 481 h^–1^ ^31^. It is found that CO_2_ can be activated into CO^*^ on the isolated Ir atom, and then couples with the methoxide adsorbed on the In_2_O_3_ to form C–C bond. Zhang and co-workers fabricated a stable Ir-based SAC for CO_2_ hydrogenation to formate. Detailed characterizations and theoretical calculations indicate that such Ir-based SAC has a similar structure with a homogeneous mononuclear Ir pincer complex catalyst, which can serve as an efficient catalyst for the conversion of CO_2_ to formate^32^. Despite this progress, CO_2_ hydrogenation to value-added liquid products over SACs is still far away from the satisfaction of chemical industry because of the following issues: (1) it is challenging to precisely regulate the selectivities of products during CO_2_ hydrogenation over SACs, (2) the poor stability of SACs at high temperature and pressure needs to be overcome, and (3) the intrinsic structure-performance relationship of SACs has not been clearly understood yet. It is thus highly desired to develop efficient SACs with enhanced activity, selectivity and stability for CO_2_ hydrogenation under mild conditions.

In this work, we used C_3_N_4_, a functional support with abundant N sites, to anchor Cu single sites for CO_2_ hydrogenation at a low temperature range of 70–150 °C. By simply altering the treatment conditions, the coordination structures of Cu single atoms can be regulated, for instance, Cu–N_4_ and Cu–N_3_ structures. Experimental results imply that Cu–N_3_ SAC displays a CO selectivity of 94.3%, while Cu–N_4_ SAC exhibit a CH_3_OH selectivity of 95.5% at a CH_3_OH productivity of 4.2 mmol g^–1^ h^–1^ at 150 °C. Impressively, the CH_3_OH productivity for Cu–N_4_ SAC has surpassed the state-of-the-art Cu-ZnO/Al_2_O_3_ by 3.2 times (1 mmol g^–1^ h^–1^). Moreover, Cu–N_4_ SAC shows promising stability without decay of CH_3_OH productivity and changes in the structure after 5 consecutive cycles. Further studies imply that CO_2_ hydrogenation follows the formate pathway to form CH_3_OH on Cu–N_4_ SAC and the RWGS pathway to form CO on Cu–N_3_ SAC, respectively. This work not only provides an efficient SAC for CO_2_ hydrogenation to form CH_3_OH under mild conditions, but also sheds interesting light on studying the structure-performance relationship of catalyst.

## Results and discussion

### Structure analysis of catalysts

**Fig. 1:**
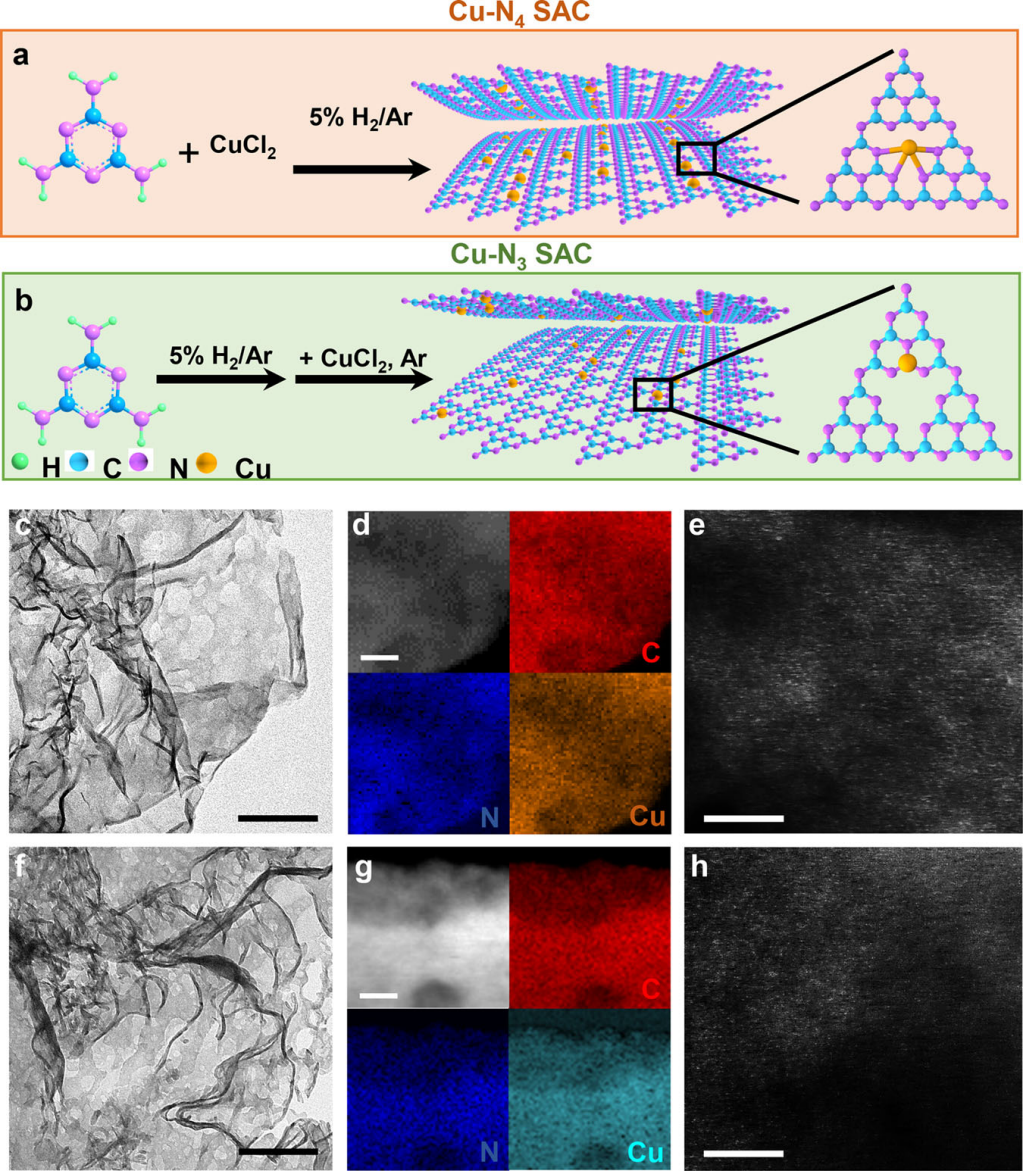
Scheme for the synthesis and characterizations of Cu–N_4_ and Cu–N_3_ SACs. Scheme for the synthesis of (**a**) Cu–N_4_ and (**b**) Cu–N_3_ SACs. **c** TEM image, (**d**) element mapping images, and (**e**) magnified HAADF-STEM image of Cu–N_4_ SAC. **f** TEM image, (**g**) element mapping images, and (**h**) magnified HAADF-STEM image of Cu–N_3_ SAC. The scale bars in (**c**–**h**) are 200, 20, 2, 200, 20, and 2 nm, respectively.

C_3_N_4_ supported Cu SACs were synthesized via the thermal pyrolysis of melamine (see experimental section for details). The physicochemical properties of catalysts including the compositions (determined by inductively coupled plasma-atomic emission spectroscopy (ICP-AES) measurement) are listed in Supplementary Table 1. Results from the N_2_ adsorption–desorption measurement suggest the generation of mesopores in Cu–N_4_ and Cu–N_3_ SACs. Consequently, the surface areas of pristine C_3_N_4_, Cu–N_4_ SAC and Cu–N_3_ SAC are 0.70, 22.5, and 20.8 m^2^/g, respectively (Supplementary Fig. 1). Transmission electron microscopy (TEM) image shows that the as-prepared C_3_N_4_ displays a morphology of nanosheet (Supplementary Fig. 2). C_3_N_4_ supported Cu SACs with tailored coordination environment, namely, Cu–N_4_ and Cu–N_3_ SACs, were generated by depositing CuCl_2_ onto C_3_N_4_ under specific conditions (Fig. 1a, b). After loading Cu on C_3_N_4_, no obvious changes in the morphologies of Cu–N_4_ and Cu–N_3_ SACs are observed (Fig. 1c, f). Scanning transmission electron microscopy energy-dispersive X-ray spectroscopy (STEM-EDS) images indicate that Cu, C, and N are well dispersed in the Cu–N_4_ and Cu–N_3_ SACs (Fig. 1d, g). Moreover, the atomic ratios of Cu in Cu–N_4_ and Cu–N_3_ SACs are 12.1% and 13.1%, respectively (Supplementary Fig. 3), which are close to those values from ICP-AES measurement (Supplementary Table 1). Atomic force microscopy (AFM) images demonstrate that Cu–N_4_ and Cu–N_3_ SACs have similar thicknesses with C_3_N_4_ (~1.6 nm, Supplementary Fig. 4). Note that the decrease of thickness of Cu–N_4_ and Cu–N_3_ SACs compared with the pristine C_3_N_4_ might be attributed to the delamination of C_3_N_4_ during the synthesis of SACs. Moreover, spherical aberration TEM (SA-TEM) measurement was employed to study the states of Cu atoms in different samples. It is found that Cu atoms, which are presented as dense white dots, display as single state in the Cu–N_4_ and Cu–N_3_ SACs (Fig. 1e, h). On the other hand, Cu NPs with a mean size of 3 nm are observed in the C_3_N_4_ supported Cu nanoparticles (Cu NPs/C_3_N_4_) (Supplementary Fig. 2b). The structures of Cu–N_4_ and Cu–N_3_ SACs were studied by X-ray diffraction (XRD) measurement. In the XRD pattern of C_3_N_4_, typical peaks appear at 2θ of 13.1° and 27.3°, which are ascribed to (100) and (002) facet, respectively (black curve in Supplementary Fig. 5). No obvious differences in the XRD patterns of Cu–N_4_ and Cu–N_3_ SACs are attributed to the isolated state of Cu atom (red and blue curves in Supplementary Fig. 5). By contrast, the typical peaks of Cu NPs appear at 43.3°, 50.4°, and 74.1° in the XRD pattern of Cu NPs/C_3_N_4_, which correspond to the characteristic peaks of Cu (111), (200), and (220) facets, respectively (Supplementary Fig. 6). Moreover, the peaks at 3145, 1639, 1145, and 798 cm^–1^ in the Fourier transform infrared spectroscopy (FTIR) spectrum of C_3_N_4_ can be indexed as the stretching vibrations of triazine ring, –NH_2_, –OH, and the bending vibration of triazine ring, respectively, and the presence of characteristic peaks of C_3_N_4_ in the FTIR spectra of Cu–N_4_ and Cu–N_3_ SACs suggested that the structure of C_3_N_4_ is largely maintained after Cu loading (Supplementary Fig. 7).

**Fig. 2:**
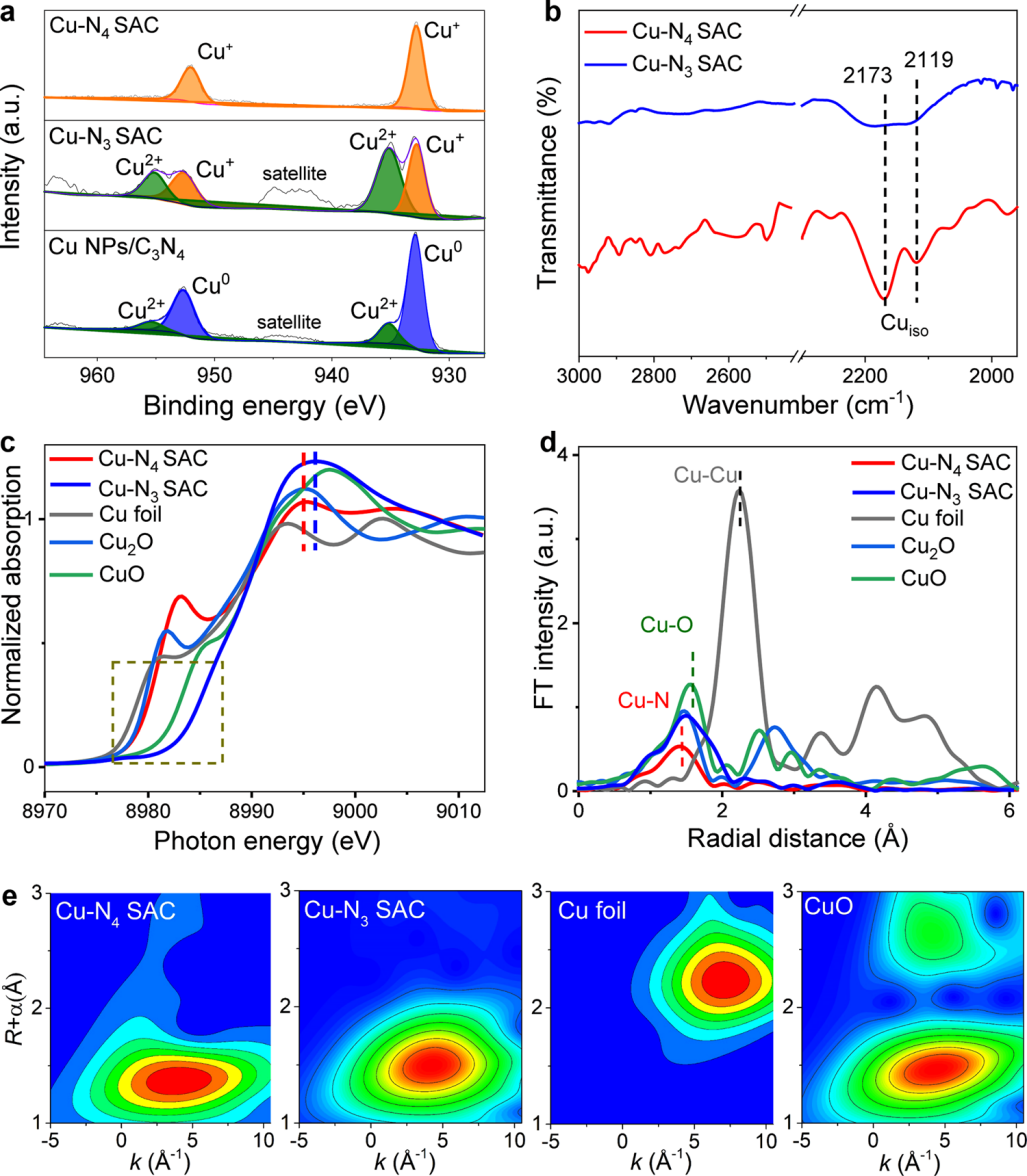
Structural characterizations of various catalysts. **a** XPS spectra of Cu–N_4_ SAC, Cu–N_3_ SAC, and Cu NPs/C_3_N_4_. **b** CO-DRIFT spectra of Cu–N_4_ and Cu–N_3_ SACs. **c** XANES and (**d**) EXAFS spectra of Cu–N_4_ SAC, Cu–N_3_ SAC, Cu foil, Cu_2_O, and CuO at Cu *K*-edge. **e** Wavelet transform of EXAFS spectra at Cu *K*-edge. Source data are provided as a Source Data file.

X-ray photoelectron spectroscopy (XPS) measurement was further used to reveal the surface properties of SACs. It is found that the surface of Cu NPs is partially oxidized, as presented by the shoulders at 934.7 and 955.0 eV in the XPS spectrum of Cu NPs/C_3_N_4_ (Fig. 2a). Considering the appearance of Cu peaks in the XRD pattern of Cu NPs/C_3_N_4_ (Supplementary Fig. 6), the peaks at 932.5 and 952.2 eV are ascribed to 2*p*_1/2_ and 2*p*_3/2_ peaks of Cu^0^. For Cu–N_4_ SACs, the characteristic peaks of Cu^+^ 2*p*_2/3_ and 2*p*_1/2_ appear at 932.5 and 952.2 eV, respectively (Note that the peaks of Cu^0^ and Cu^+^ locates at similar positions in XPS spectrum). For Cu–N_3_ SACs, in addition to the features of Cu^+^, two peaks corresponding to Cu^2+^ appear at 935.0 and 955.0 eV in the Cu 2*p* XPS spectrum with a Cu^2+^/Cu^+^ ratio of ~2 (Fig. 2a). For N 1*s* XPS spectra of Cu–N_4_ and Cu–N_3_ SACs, the features of pyridinic N, pyrrolic-N and graphic-N are observed at 398.6, 400.2 and 401.3 eV, respectively, which are similar to those of C_3_N_4_ (Supplementary Fig. 8). Moreover, the oxidation states of Cu in Cu–N_4_ and Cu–N_3_ SACs have been further confirmed by Auger Electron Spectroscopy (AES) spectra, in which the peaks negatively shift to lower binding energies compared with Cu NPs/C_3_N_4_ (Supplementary Fig. 9). To further reveal the chemical states of Cu atoms in Cu–N_4_ and Cu–N_3_ SACs, diffuse reflectance infrared Fourier transform spectroscopy (DRIFTS) measurement using CO as the probe molecule was performed. Two weak bands appear at 2173 and 2119 cm^–1^ in the CO-DRIFTS spectra of Cu–N_4_ and Cu–N_3_ SACs, which are ascribed to CO linear adsorption on Cu atoms (Fig. 2b). Additionally, X-ray absorption near-edge structure (XANES) and extended X-ray absorption fine structure (EXAFS) measurement were performed at Cu K-edge to investigate the local structures of Cu atoms in catalysts. Compared to Cu foil, the absorption edges for Cu–N_4_ and Cu–N_3_ SACs shift to higher energy, suggesting the oxidation states of Cu in Cu–N_4_ and Cu–N_3_ SACs (Fig. 2c). Moreover, we estimated the mean chemical valences of Cu in Cu–N_4_ and Cu–N_3_ SACs based on the XANES spectra of Cu–N_4_ SAC, Cu–N_3_ SAC, Cu foil, Cu_2_O, and CuO. As shown in Supplementary Fig. 10, the mean chemical valences of Cu in Cu–N_4_ and Cu–N_3_ SACs are +1.05 and +1.64, respectively. In the EXAFS spectra of Cu_2_O and CuO, the peaks at 1.55 and 2.23 Å are ascribed to the coordination of Cu–O and Cu–Cu, respectively (Fig. 2d). The absences of Cu–Cu coordination in the EXAFS spectra of Cu–N_4_ and Cu–N_3_ SACs suggest that Cu atoms are presented as isolated state (Fig. 2d and Supplementary Table 2). Additionally, wavelet transform (WT) at the Cu K-edge EXAFS analysis further confirms the isolated state of Cu atoms in Cu–N_4_ and Cu–N_3_ SACs. As shown in Fig. 2e, the intense Cu–N coordination, which is illustrated by red color, is observed in the WT contour plots of Cu–N_4_ SAC and Cu–N_3_ SAC at the radial distances of ~1.45 Å (Y axis). Also, the strong Cu–Cu (~2.2 Å) and Cu–O (1.5 Å) coordination can be observed in the WT contour plots of Cu foil and CuO, which agrees with the results from EXAFS spectra (Fig. 2e).

### CO_2_ hydrogenation

**Fig. 3:**
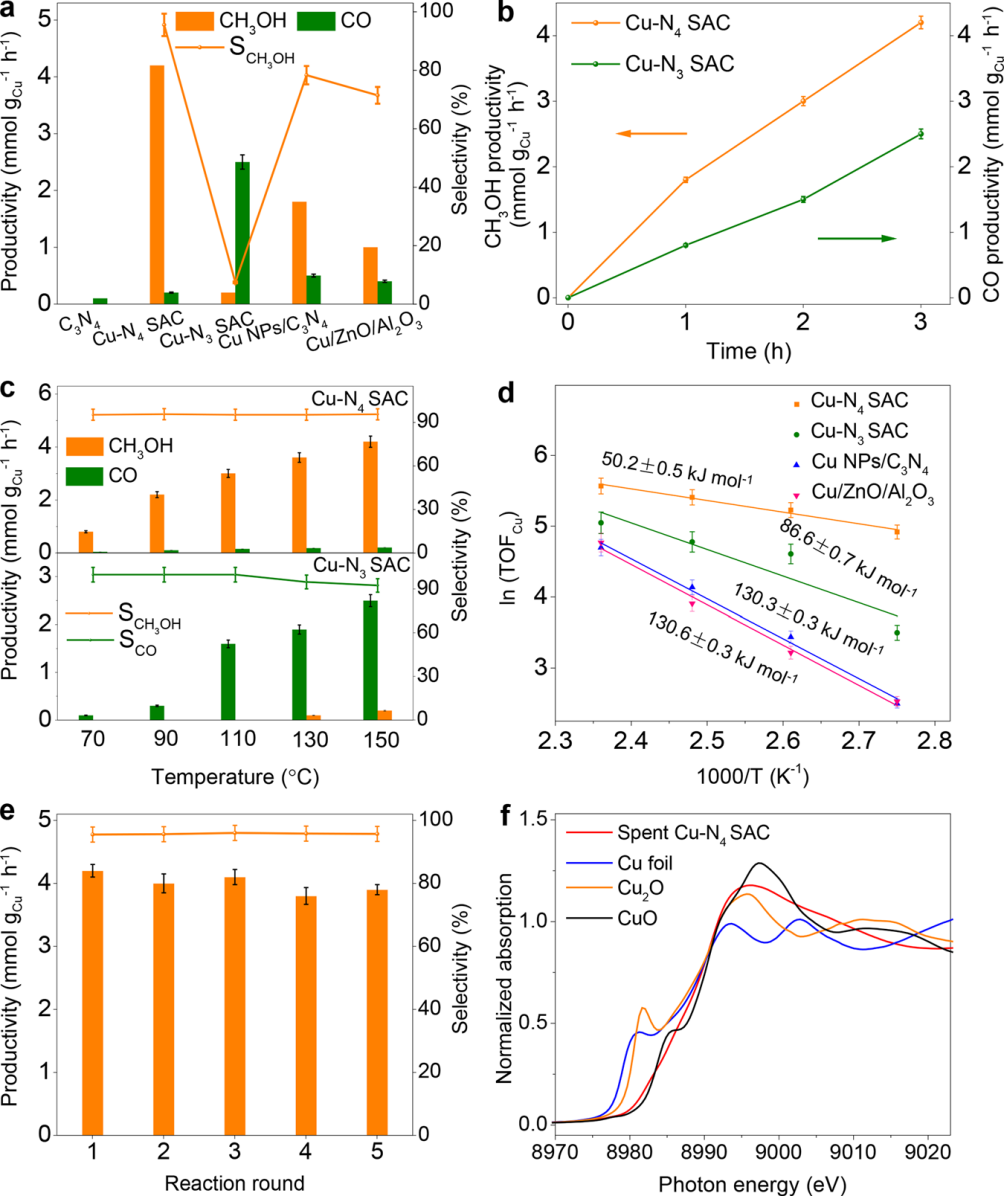
Catalytic performance of CO_2_ hydrogenation. **a** Catalytic performance of CO_2_ hydrogenation over different catalysts. **b** CH_3_OH and CO productivities of Cu–N_3_ SAC and Cu–N_4_ SAC, respectively. Reaction conditions for (**a**) and (**b**): H_2_/CO_2_/N_2_ = 72:24:4 vol.%, *P*_total_ = 3.2 MPa, *T* = 150 °C, and reaction time is 3 h. **c** CO_2_ hydrogenation over Cu–N_4_ and Cu–N_3_ SACs at different temperatures. Reaction conditions: H_2_/CO_2_/N_2_ = 72:24:4 vol.%, *P*_total_ = 3.2 MPa, *T* = 70–150 °C, and reaction time is 3 h. **d** Arrhenius plots of different catalysts. **e** CH_3_OH productivity and selectivity over Cu–N_4_ SAC in 5 cycles. Reaction conditions: H_2_/CO_2_/N_2_ = 72:24:4 vol.%, *P*_total_ = 3.2 MPa, *T* = 150 °C, and the reaction time for each cycle is 3 h. **f** XANES spectra of the spent Cu–N_4_ SAC. All the data in (**a**–**e**) were collected for three times, and the error bars represent the standard deviation. Source data are provided as a Source Data file.

The performance of CO_2_ hydrogenation was evaluated in a batch stainless-steel reactor with 10 mg catalyst and 10 mL water. Commercial Cu-ZnO/Al_2_O_3_, C_3_N_4_, and Cu NPs/C_3_N_4_ were used as references. Prior to catalytic tests, the reactor was pre-flushed with N_2_ for five times. Afterwards, a mixture of H_2_, CO_2_, and N_2_ (72:24:4 vol.%) was pressurized into the reactor with a total pressure of 3.2 MPa. As shown in Fig. 3a, no gaseous (e.g., CO and CH_4_) and liquid products (e.g., CH_3_OH) were detected at 150 °C when C_3_N_4_ was as catalyst, indicating that C_3_N_4_ is inactive under the indicated conditions. When Cu NPs/C_3_N_4_ was used as catalyst, CH_3_OH productivity is 1.8 mmol g^–1^ h^–1^ at a CH_3_OH selectivity of 78.6%. CH_3_OH productivity significantly increases to 4.2 mmol g^–1^ h^–1^ at a CH_3_OH selectivity of 95.5% for Cu–N_4_ SAC. In contrast, CO selectivity reaches 94.3% with a CO productivity of 2.5 mmol g^–1^ h^–1^ when Cu–N_3_ SAC was used as catalyst. It is noted that the productivity and selectivity of CH_3_OH for Cu–N_4_ SAC are much higher than those of Cu NPs/C_3_N_4_ (Supplementary Fig. 11) and commercial Cu-ZnO/Al_2_O_3_ (Supplementary Fig. 12). Moreover, CH_3_OH and CO productivities reach 4.2 and 2.5 mmol g^–1^ h^–1^, respectively, when the reaction time was extended to 3 h. As shown in Fig. 3c, when Cu–N_4_ SAC was used as catalyst, the CH_3_OH productivity increases from 0.6 to 4.2 mmol g^–1^ h^–1^ with the increased temperature from 70 to 150 °C, whereas the selectivity of CH_3_OH is kept at ~95%. On the other hand, the CO productivity increases from 0.2 to 2.6 mmol g^–1^ h^–1^ for Cu–N_3_ SAC when the reaction temperature was elevated from 70 to 150 °C (Fig. 3c). We also evaluated the catalytic performance at the temperature range from 70 to 150 °C (Supplementary Figs. 13, 14). It is found high temperature favors CO_2_ hydrogenation over Cu–N_4_ (to produce CH_3_OH) and Cu–N_3_ (to produce CO) SACs. The above results indicate that Cu–N_4_ SAC can serve as highly active and selective catalyst for CO_2_ hydrogenation to CH_3_OH, while Cu–N_4_ SAC favors for CO formation (Fig. 3b). Moreover, the kinetics for CO_2_ hydrogenation were studied for further evaluating the catalytic performance. Arrhenius plots indicate that the apparent activation energies (Ea) for the commercial Cu-ZnO/Al_2_O_3_, Cu NPs/C_3_N_4_, Cu–N_4_ SAC, and Cu–N_3_ SAC are 130.6, 130.3, 50.2, and 86.6 kJ mol^–1^, respectively (Fig. 3d). The smallest value of Ea for Cu–N_4_ SAC indicates that Cu–N_4_ SAC can serve as the most active catalyst for CO_2_ hydrogenation, which is consistent with our experimental observations. We further calculated the reaction orders with respect to CO_2_ and H_2_ for Cu–N_4_ SAC, and the reaction orders of CO_2_ and H_2_ are 1.18 and 1.19, respectively (Supplementary Fig. 15). Note that the catalytic performance of Cu–N_4_ SAC has surpassed most of the reported Cu-based catalysts for CO_2_ hydrogenation (Supplementary Table 3).

Additionally, the stability of Cu–N_4_ SAC was evaluated in five consecutive cycles at 150 °C. No obvious decay of the CH_3_OH productivity indicates the promising stability of Cu–N_4_ SAC for CO_2_ hydrogenation (Fig. 3e). Besides, the local structures of Cu atoms are largely maintained for the spent Cu–N_4_ SAC (Fig. 3f, Supplementary Figs. 16, 17). ICP-AES results indicate that the content of Cu in spent Cu–N_4_ SAC is close to that of fresh Cu–N_4_ SAC (10.2%), confirming the enhanced stability of Cu–N_4_ SACs for CO_2_ hydrogenation. Additionally, in situ IR (Supplementary Fig. 18) and XRD measurements were performed for Cu–N_4_ and Cu–N_3_ SACs for further studying the stability of Cu–N_4_ SAC for CO_2_ hydrogenation. No obvious changes are observed in the IR spectra collected at 3 MPa and 150 °C with different reaction times, suggesting the promising stabilities of catalysts for CO_2_ hydrogenation (Supplementary Fig. 19). In particular, the peak at 3170, 1650, 1175 and 808 cm^-1^ are ascribed to the vibrations of triazine ring stretching, –NH_2_, –OH stretching, and triazine ring bending, respectively. Besides, in situ XRD patterns were collected at different temperature and in the presence of H_2_/CO_2_/N_2_ (72:24:4 vol.%) with a total pressure of 0.03 MPa. As shown in Supplementary Fig. 20, the peak at 27.3° can be indexed as the (002) facet of C_3_N_4_, and no obvious changes were observed when the temperature was increased from 40 to 150 °C, further confirming the enhanced stability of Cu–N_4_ SAC. Note that the slight decrease of productivity in 5 cycles might be attributed to the catalyst loss during centrifugation.

### Catalysis mechanism studies

To understand the distinct performance and catalysis mechanism of Cu–N_4_ and Cu–N_3_ SACs towards CO_2_ hydrogenation, in situ DRIFTS measurement was performed by exposing different catalysts to a mixture of CO_2_ and H_2_ (1:3 vol.%) at 0.1 MPa and 150 °C. No peaks were observed in the DRIFTS spectra before introducing CO_2_ and H_2_ onto Cu–N_4_ and Cu–N_3_ SACs (red curves in Supplementary Fig. 21a, b). After the introduction of CO_2_ and H_2_, several peaks appear at 3265, 2964, 2848, 2514, 2014, and 1917 cm^–1^ in the DRIFTS spectrum of Cu–N_4_ SAC, which can be indexed as the characteristic peaks of CH_3_O^*^ (Supplementary Fig. 21a). In contrast, two weak peaks are observed at 2114 and 2176 cm^–1^ in the DRIFTS spectrum of Cu–N_3_ SAC, which are ascribed to CO^*^ adsorption (Supplementary Fig. 21b). Additionally, given the significant differences in the coordination environment of Cu–N_4_ SAC (consists of Cu^+^) and Cu–N_3_ SAC (consists of Cu^+^ and Cu^2+^), we further studied the effects of Cu^2+^/Cu^+^ ratio on the performance of CO_2_ hydrogenation. Considering H_2_ can reduce Cu^2+^ into Cu^+^, we thus treated Cu–N_3_ SAC at 500 °C for different times. In particular, the Cu^2+^/Cu^+^ ratios are 1 and 0.5 when Cu–N_3_ SAC was treated at 500 °C for 15 and 30 min, respectively. When the Cu^2+^/Cu^+^ ratio is increased from 0.5 to 2 (Supplementary Fig. 22), CH_3_OH productivity significantly decreases from 2.8 to 0.25 mmol g^–1^ h^–1^, while CO productivity significantly increases from 0.5 to 2.6 mmol g^–1^ h^–1^ (Supplementary Fig. 23). The aforementioned results imply that the catalytic performance of CO_2_ hydrogenation was strongly depended on the coordination environment of Cu atoms.

**Fig. 4:**
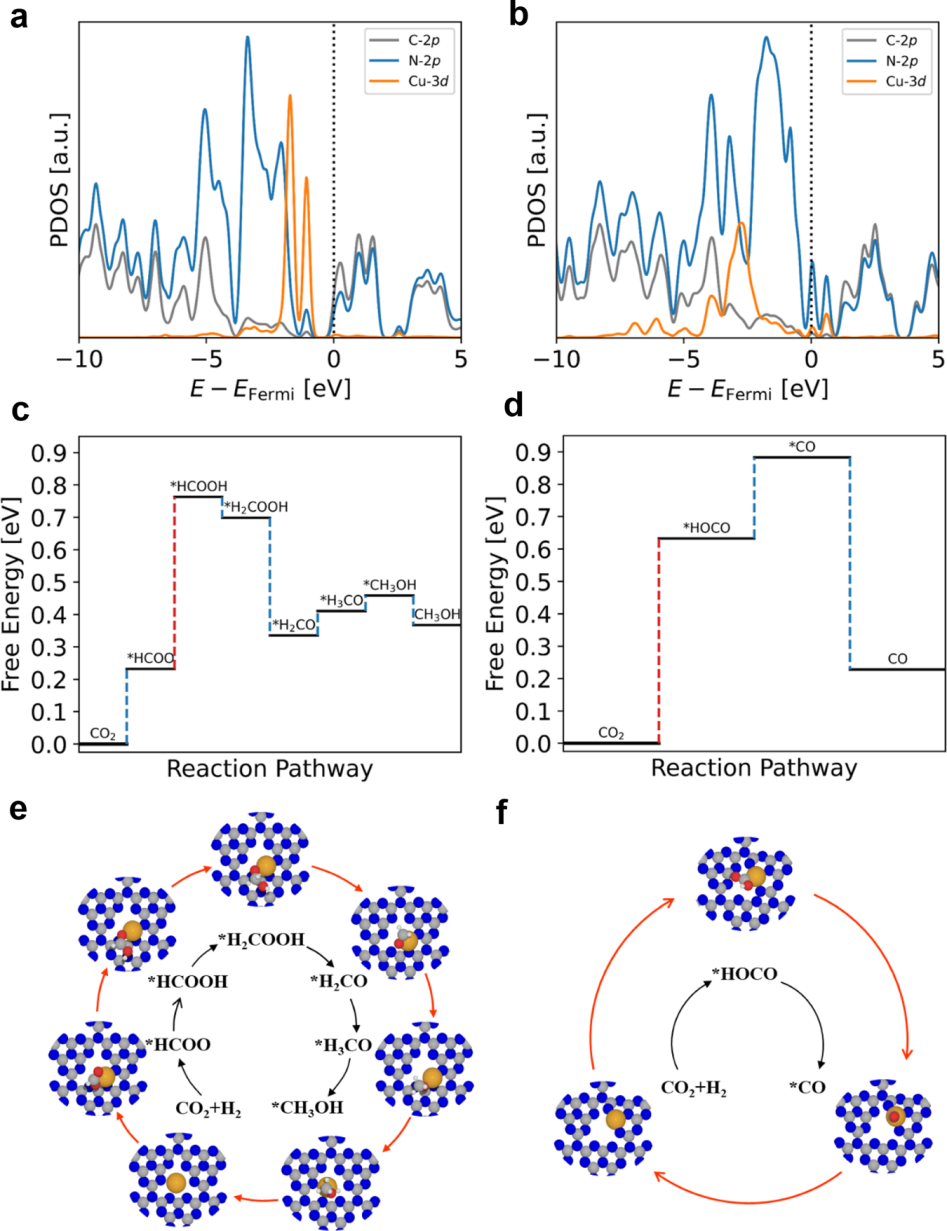
Theoretical calculations and possible reaction paths for CO_2_ hydrogenation. Projected density of states for (**a**) Cu–N_4_ and (**c**) Cu–N_3_ SACs, and the Fermi level is indicated by the dashed line. Energy profiles for (**b**) CO_2_ hydrogenation via formate pathway over Cu–N_4_ SAC and (**d**) CO_2_ hydrogenation via RWGS pathway over Cu–N_3_ SAC. Reaction pathways and corresponding atomic configurations for CO_2_ hydrogenation to (**e**) CH_3_OH and (**f**) CO. The temperature is set as 423 K. The yellow, blue, gray, red and white balls in (**e**) and (**f**) represent as Cu, N, C, O, and H atoms, respectively.

Density functional theory (DFT) calculations were further performed to confirm the stable configurations of Cu single atoms on C_3_N_4_ and the corresponding mechanism for catalysis. We choose the buckled C_3_N_4_ structure as the substrate, because it is more stable than the planar one, which has been fully discussed in the previous studies^33–35^. EXAFS analysis indicates that the coordination numbers of Cu–N for Cu–N_3_ and Cu–N_4_ SACs are ~3.1 and 3.7, respectively (Supplementary Table S2), we thus selected two models to mimic the structures of Cu single atoms on C_3_N_4_: 1) Cu atom locates at the hole site of C_3_N_4_ (Supplementary Fig. 24). After structural relaxation, Cu atom will not locate at the center of the hole but slightly moves to one side. Cu atom strongly coordinates with two N atoms with the bond length of 2.18 Å and 2.16 Å, and two longer Cu–N bonds of 2.28 Å and 2.38 Å are also formed. In this model, Cu atom is coordinated with 4 N atoms to form structure of Cu–N_4_, which is consistent with the results from EXAFS measurement. The adsorption energy of Cu is 2.98 eV, which is higher than the adsorption energy of Pd single atoms on C_3_N_4_ (2.17 eV) in previous work^36^, indicating that Cu atoms can be firmly anchored on C_3_N_4_ in single state; 2) Cu atom replaces one of C atoms in C_3_N_4_ to form three Cu–N bonds (Supplementary Fig. 25). In this model, Cu is coordinated with 3 N atoms, and the length of Cu–N bond is as short as ~1.80 Å, indicating a strong coordination between Cu and N atoms. To confirm the thermodynamic stability of our two models, we have performed the molecular dynamics (MD) calculations at the temperature of 450 K for totally 10 ps with a time step of 1 fs for both Cu–N_3_ and Cu–N_4_, respectively. From our results, we can observe the fluctuation of the total energy with the time evolution, as shown in Supplementary Fig. 26. The energies are oscillating near the equilibrium state and there is no structural disruption throughout simulations, indicating the two models are thermodynamically stable. Furthermore, we estimated the Bader charges of Cu in the above two models. It is found that the Bader charge of Cu in Cu–N_3_ model is +0.95e, which is larger than that of Cu–N_4_ model (+0.73e), suggesting that more electrons transfer from Cu to neighboring N atoms in Cu–N_3_ model compared to Cu–N_4_ model (Supplementary Fig. 27). Consequently, the valence of Cu in Cu–N_3_ is higher than that in Cu–N_4_, which is consistent with the results from XANES measurement. Additionally, we analysed the projected density of states (PDOS) of Cu in Cu–N_3_ and Cu–N_4_ models to study the electronic structures. It is found that the *3d* orbital of Cu atom strongly couples with the *2p* orbital of N atom, which confirms the strong coordination between Cu single atoms and C_3_N_4_ (Fig. 4a, b). Compared to Cu–N_4_ ( + 0.73e), the orbital coupling between Cu and N atoms for Cu–N_3_ (+0.95e) shifts to lower energy levels (far away from the Fermi level), indicating a relatively higher valence state of Cu, which is in agreement with characterizations and Bader charge analysis. We finally simulated the *K*-edge XANES spectra of Cu–N_4_ and Cu–N_3_ SACs based on the selected models. As shown in Supplementary Fig. 28, it is found that both the simulated XANES spectra of Cu–N_4_ and Cu–N_3_ models display similar features with those of the original ones, further indicating that the selected Cu–N_4_ and Cu–N_3_ models are representative for investigating the mechanism for catalysis.

Moreover, DFT calculations were conducted to investigate the reaction pathways of CO_2_ hydrogenation on Cu–N_4_ and Cu–N_3_ SACs. Based on the previous reports^19,20^, the major reaction pathway for CO formation is the reverse-water-gas-shift (RWGS) reaction via carboxyl (^*^HOCO) intermediate, while the formation of CH_3_OH involves a formate pathway. We then systematically investigated all these possible pathways on Cu–N_4_ and Cu–N_3_ SACs. On Cu–N_4_, DFT calculations imply that the first H prefers to bond with C atom of CO_2_ to form ^*^HCOO, instead of forming the ^*^HOCO intermediate (the free energy for ^*^HOCO formation is higher than that for ^*^HCOO formation by 0.30 eV at the experimental temperature). ^*^HCOO intermediate then undergoes hydrogenation to form ^*^HCOOH and ^*^H_2_COOH. The formation of ^*^H_2_CO is energetically allowed through the cleavage of C − O bond in ^*^H_2_COOH with the free energy decrease by 0.36 eV (Fig. 4c). In contrast, DFT calculations indicate that the formation of ^*^HCOO and ^*^HOCO completely occurs on Cu–N_3_ SAC, and the free energies for ^*^HCOO and ^*^HOCO are 0.59 and 0.63 eV, respectively (Fig. 4d). However, the formation of ^*^H_2_COOH via ^*^HCOOH hydrogenation is not allowed by giving a high free energy barrier of 0.75 eV. Consequently, ^*^HOCO is converted into CO via the RWGS pathway. On the other hand, the adsorption energy of CO on Cu–N_3_ SAC is only –0.36 eV, indicating that CO can desorb from the surface of catalyst with a free energy decrease of 0.66 eV (Fig. 4d). Based on the aforementioned results, we conclude that CO_2_ hydrogenation follows the formate pathway to form CH_3_OH on Cu–N_4_ SAC (Fig. 4e) and the RWGS pathway to form CO in Cu–N_3_ SAC (Fig. 4f).

In summary, we have synthesized C_3_N_4_ supported Cu SAC with a high loading amount of 12 wt.% for low temperature CO_2_ hydrogenation. By simply altering the parameters for treatment, the coordination structure of Cu atoms in SAC can be systematically tuned by altering the conditions for treatment (e.g., Cu–N_4_ and Cu–N_3_), which leads to the significant differences in the CO_2_ hydrogenation. Mechanism studies suggest that the reaction pathways are significantly related to the coordination structure of Cu atoms. In particular, Cu–N_4_ SAC exhibits a CH_3_OH productivity of 4.2 mmol g^–1^ h^–1^ at a CH_3_OH selectivity of 95.5% at 150 °C, which has surpassed that of commercial Cu/ZnO/Al_2_O_3_ by 3.2 times (1 mmol g^–1^ h^–1^). Detailed investigations show that CO_2_ hydrogenation follows the formate pathway to form CH_3_OH on Cu–N_4_ SAC and the RWGS pathway to form CO on Cu–N_3_ SAC, respectively. This work not only provides an active and selective SAC for low temperature CO_2_ hydrogenation, but also sheds lights on studying the structure**-**performance relationship of catalyst.

## Methods

### Chemicals

Copper (II) chloride dihydrate (CuCl_2_.2H_2_O, > 99.9%) and melamine were purchased from Shanghai Chemical Reagent company. All chemical reagents were used without further purification. All aqueous solutions were prepared using deionized water with a resistivity of 18.2 MΩ cm^–1^.

### Synthesis of C_3_N_4_

In a typical preparation of C_3_N_4_, 500 mg melamine was heated to 550 °C with a rate of 8 °C min^–1^ in a tube furnace under 5% H_2_/Ar atmosphere for 4 h. After cooling to room temperature, the products were grinded and collected.

### Synthesis of Cu–N_4_ SAC

In a typical synthesis of Cu–N_4_ SAC, 500 mg melamine, 200 mg CuCl_2_, 5 mL ethanol and 5 mL water were added into a 30 mL vial. The mixture was stirred for 24 h and then centrifuged. The obtained sample was dried in a vacuum oven at 60 °C for 24 h and was thoroughly grinded. Finally, the sample was heated to 550 °C at 8 °C min^–1^ and maintained at the temperature for 4 h in H_2_/Ar flow (5 vol.% H_2_ in Ar). Afterwards, the sample was collected after cooling to room temperature.

### Synthesis of Cu–N_3_ SAC

For the synthesis of Cu–N_3_ SAC, 100 mg C_3_N_4_ and 23 mg CuCl_2_ were added into 20 mL ethanol under moderate stirring for 12 h at 80 °C. After that, the product was centrifuged and washed with distilled water and ethanol for three times, followed by drying in vacuum at 70 °C for 24 h. Finally, the sample was heated at 500 °C for 2 h in Ar flow to obtain Cu–N_3_ SAC.

### Synthesis of Cu NPs/C_3_N_4_

For the synthesis of Cu NPs/C_3_N_4_, the parameters were same with those of C_3_N_4_ supported Cu SACs, except for the final treatment in 0.5 M NaBH_4_ for 30 min at room temperature.

### Characterizations

X-ray diffraction (XRD) measurement was conducted on an X’Pert-Pro X-ray powder diffractometer equipped with a Cu radiation source (*λ* = 0.15406 nm). High resolution transmission electron microscopy and elemental line scans were performed on a JEOL-2100F transmission electron microscope at an acceleration voltage of 200 kV. High**-**angle annular dark-field STEM energy-dispersive X-ray spectroscopy (HAADF-STEM-EDS) were conducted on a JEOL GrandARM300F scanning transmission electron microscope with double Cs correctors at an acceleration voltage of 300 kV. Raman spectra were recorded on a HORIBA HR800 instrument. AFM was collected on a Bruker Dimension. Attenuated total reflectance infrared (ATR-IR) spectra were recorded on a Bruker Vertex 70 spectrometer in the spectral range of 4000–400 cm^–1^. All the XPS spectra of the Cu NPs were collected by XPS (Thermo Scientific, ESCALAB 250 XI). The concentrations of all the catalysts were determined by the inductively coupled plasma-atomic emission spectroscopy (ICP-AES) (710-ES, Varian). The surface properties like Brunauer–Emmett–Teller surface area, Barrett-Joyner-Halenda (BJH) porosity, pore volume of materials was examined by N_2_ adsorption-desorption isotherms at 77 K by using Autosorb Quantachrome 1MP instrument. The in situ Fourier transform infrared (FTIR) spectra of CO adsorption were measured by a Nicolet 6700 equipped with the liquid nitrogen cooled mercury-cadmium-telluride detector (MCT). Quick-scanning X-ray absorption spectroscopy beamline using bending magnet at Taiwan Photon Source (TPS).

### Catalytic tests

The CO_2_ hydrogenation was performed in a 60 mL stainless-steel autoclave. The commercial Cu-ZnO/Al_2_O_3_ was used as a reference, which was pre-treated in 10%H_2_/Ar at 250 °C for 4 h. After the additions of 10 mL H_2_O and 10 mg catalysts into a Teflon inlet, the autoclave was pressurized with CO_2_ (0.8 MPa) and H_2_ (2.4 MPa). The reaction was performed at 150 °C with moderate magnetic stirring for 3 h. After completion of the reaction, the gaseous mixture was analyzed using a gas chromatograph (Shiweipx GC-7806) equipped with a GDX-502 column connected to a thermal conductivity detector. The liquid mixture was collected by centrifugation at 14000 × g for 3 min. 10 μL isopropanol was introduced into 1 mL reaction mixture as an internal standard. The liquid mixture was analysed using a gas chromatograph (Persee G5) equipped with a KB-5 column connected to a flame ionization detector. The tests were repeated three times for each catalyst.

### Theoretical methods

DFT calculations were performed by using the Perdew–Burke–Ernzerhof (PBE) functional^37,38^ within the generalized gradient approximation implemented in Vienna Ab Initio Simulation Package^39,40^. The projector-augmented wave method^41,42^ was applied to describe the electron–ion interactions, and the D3 Grimme’s method was employed to correct van der Waals interaction^43^. We used a plane-wave cutoff energy of 520 eV and the Gaussian smearing with a width of 0.05 eV. Periodic boundary conditions were applied, and more than 15 Å of vacuum space was used to avoid the interaction of the adjacent images. A Γ-centered (3, 3, 1) *k*-point grid was adopted to sample the Brillouin zone of a 2 × 2 buckled C_3_N_4_ supercell with the lattice constant of 6.94 Å. All the structures were fully relaxed until the force components were < 0.02 eV Å^–1^.

The free energy (G) for each species is expressed as Eq. (1):

1 $${{{{{\rm{G}}}}}}\,=\,{{{{{{\rm{E}}}}}}}_{{{{{{\rm{DFT}}}}}}}\,+\,{{{{{{\rm{E}}}}}}}_{{{{{{\rm{ZPE}}}}}}}\,-\,{{{{{\rm{TS}}}}}}$$

where E_DFT_, E_ZPE_, S, and T represents electronic energy, zero-point energy, entropy and temperature (423 K), respectively. The ideal gas approximation and the harmonic approximation were used for H_2_O, H_2_, CO_2_, CO and CH_3_OH molecules, and all atomic nuclear motions for adsorbates were considered as harmonic oscillators. The relevant contributions to *G* were listed in Supplementary Table 4. A series of gas-phase thermochemical reaction enthalpies (Supplementary Table 5) were tested to correct the DFT energies of CO_2_ and CO due to the inaccuracy of the PBE functional to describe those molecules^44^. The correction energies of +0.11 and −0.34 eV were applied for CO_2_ and CO, respectively.

XANES simulations. Cu K-edge XANES spectra were simulated on FDMNES^45^ based on the cell structure of Cu–N_4_ (Supplementary Fig. 24) and Cu–N_3_ models (Supplementary Fig. 25) used for DFT calculations. Cu reference (ICSD no. 64699) was used for calibrating energy. Hubbard energy of 5 eV was used for all the Cu K-edge simulations^46^, and the Green function employing Muffin-tin potential was applied for the XANES calculations with Athena 0.9.26^47^.

### Reporting summary

Further information on research design is available in the Nature Research Reporting Summary linked to this article.

## Supplementary information

Supplementary Information

Reporting Summary

## Data Availability

All relevant data are available from the authors on request. The theoretical calculation data generated in this study are provided in the Supplementary Information. Source data are provided with this paper.

## Source data

Source Data
