# Supplementary material for: Coordination tailoring of Cu single sites on C3N4 realizes selective CO2 hydrogenation at low temperature
Source: Nat Commun. 2021 Oct 15;12:6022. doi: 10.1038/s41467-021-26316-6 (PMC8519910; doi:10.1038/s41467-021-26316-6)
Supplement: Supplementary file 1 — Supplementary Information [file 41467_2021_26316_MOESM1_ESM.pdf]

Supplementary information

Coordination Tailoring of Cu Single Sites on C_3N_4 Realizes Selective CO_2 Hydrogenation at Low Temperature

Tang Yang[1], Xinnan Mao[2,3], Ying Zhang[1], Xiaoping Wu[3], Lu Wang[2,*], Mingyu Chu[2,3], Chih-Wen Pao[4], Shize Yang[5], Yong Xu[3,*] and Xiaoqing Huang[1,*]

[1]State Key Laboratory of Physical Chemistry of Solid Surfaces, College of Chemistry and Chemical Engineering, Xiamen University, Xiamen, 361005, China.

[2]Institute of Functional Nano & Soft Materials (FUNSOM), Jiangsu Key Laboratory for Carbon-Based Functional Materials & Devices, Soochow University, Suzhou, 215123, China.

[3]Guangzhou Key Laboratory of Low-Dimensional Materials and Energy Storage Devices, Collaborative Innovation Center of Advanced Energy Materials, School of Materials and Energy, Guangdong University of Technology, Guangzhou, 510006, China.

[4]National Synchrotron Radiation Research Center, Hsinchu, 30076, Taiwan.

[5]Eyring Materials Center, Arizona State University, Tempe, Arizona, 85287, USA.

E-mail: lwang@suda.edu.cn, yongxu@gdut.edu.cn, hxq006@xmu.edu.cn

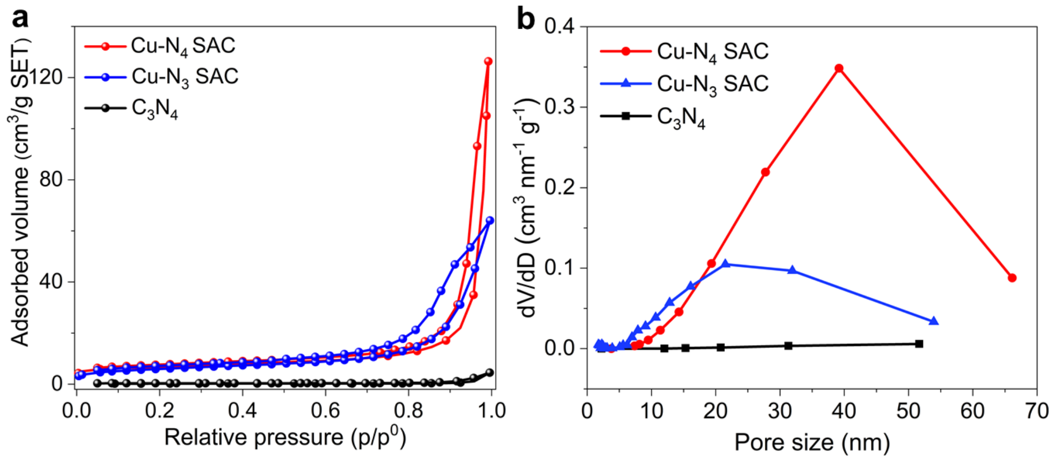

Supplementary Figure 1 | Physicochemical properties of various catalysts. (**a**) N_2 sorption isotherms and (**b**) pore size analysis of Cu–N_4 SAC, Cu–N_3 SAC and C_3N_4. Source data are provided as a Source Data file.

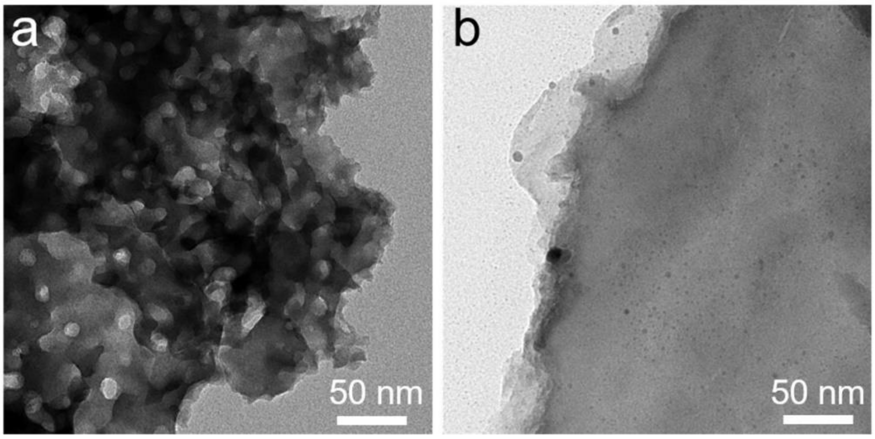

Supplementary Figure 2 | Characterizations of various samples. TEM images of (**a**) C_3N_4 and (**b**) Cu NPs/C_3N_4. Cu nanoparticles are labeled in (**b**).

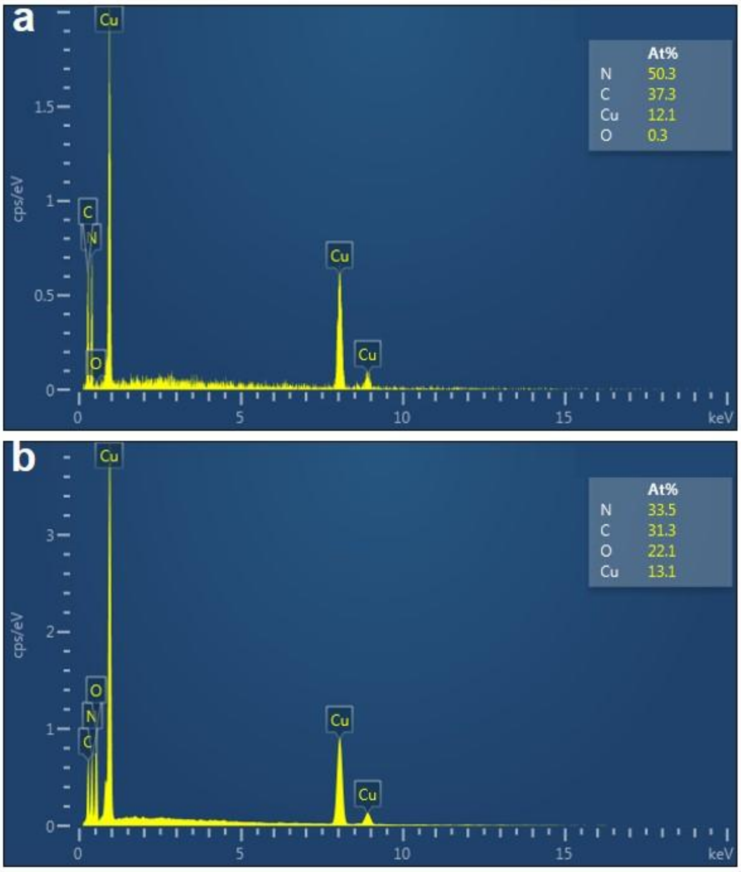

Supplementary Figure 3 | Characterizations of SACs. EDX spectrum of (**a**) Cu–N_4 SAC and (**b**) Cu–N_3 SAC.

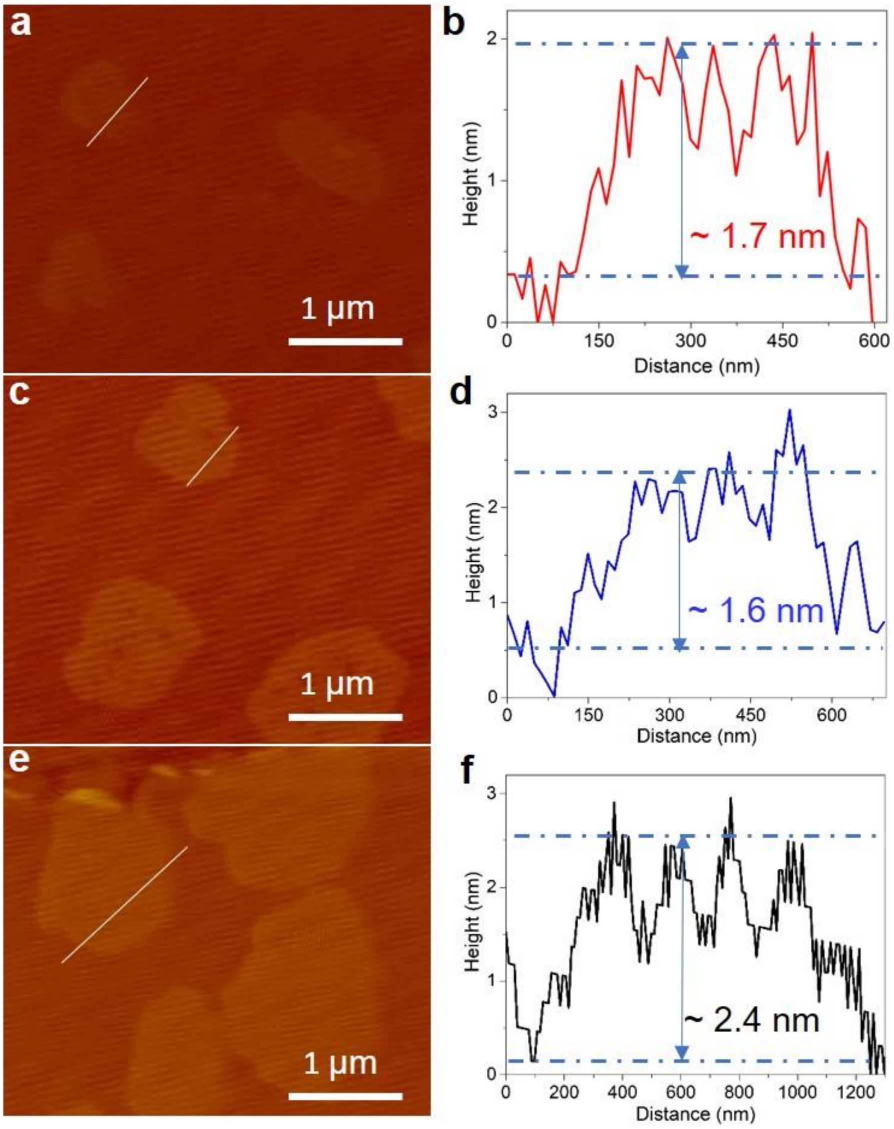

Supplementary Figure 4 | Thickness measurements of various catalysts. (**a, c, e**) AFM images and (**b, d, f**) height profiles of (**a, b**) Cu–N_4 SAC, (**c, d**) Cu–N_3 SAC and (**e, f**) C_3N_4. Source data are provided as a Source Data file.

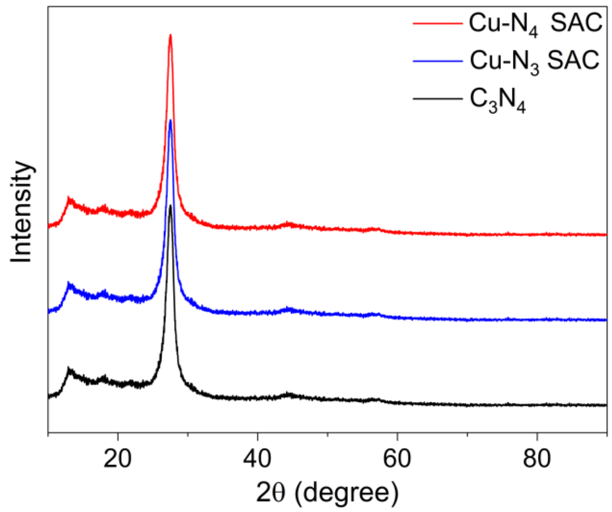

Supplementary Figure 5 | Characterizations of various catalysts. XRD patterns of Cu–N_4 SAC, Cu–N_3 SAC and C_3N_4. Source data are provided as a Source Data file.

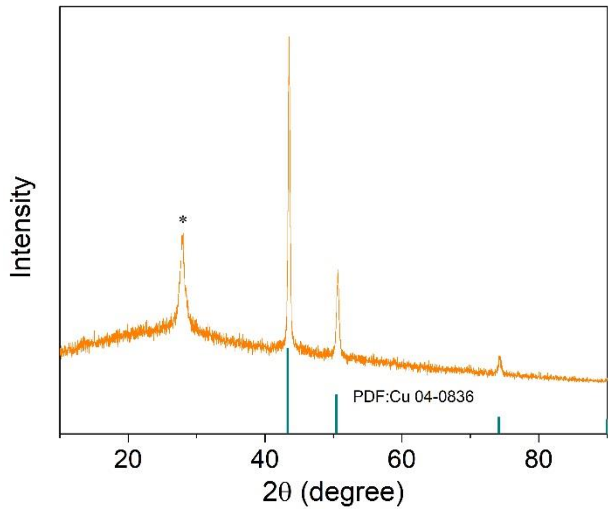

Supplementary Figure 6 | XRD pattern of Cu NPs/C_3N_4. "*" represents the characteristic peak of C_3N_4. Source data are provided as a Source Data file.

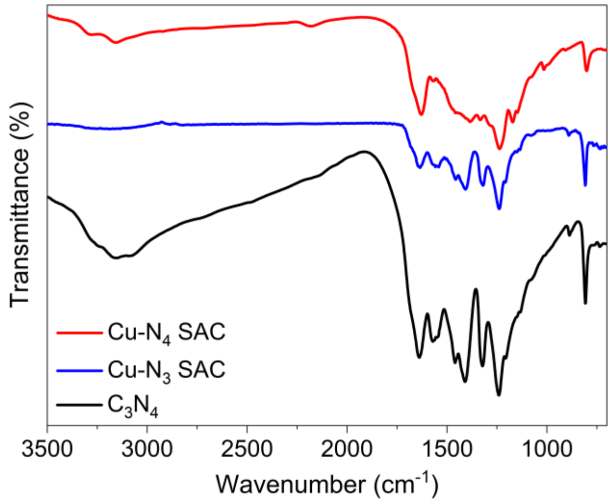

Supplementary Figure 7 | Characterizations of various catalysts. IR spectra of Cu–N_4 SAC, Cu–N_3 SAC and C_3N_4. Source data are provided as a Source Data file.

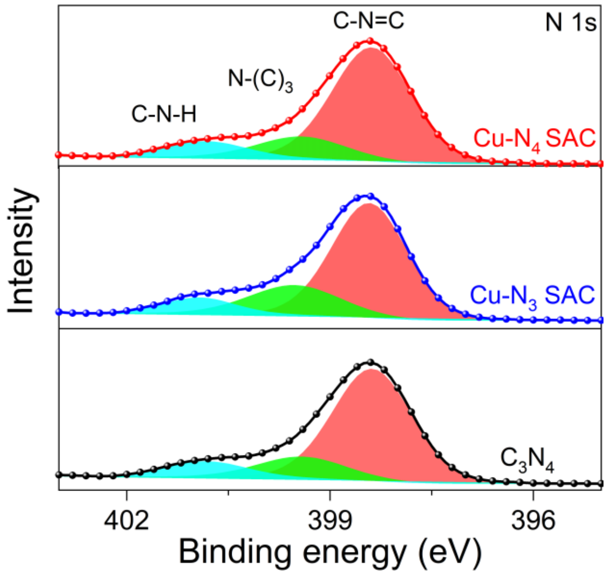

Supplementary Figure 8 | XPS spectra of various catalysts. N 1s XPS spectra of Cu–N_4 SAC, Cu–N_3 SAC and C_3N_4. Source data are provided as a Source Data file.

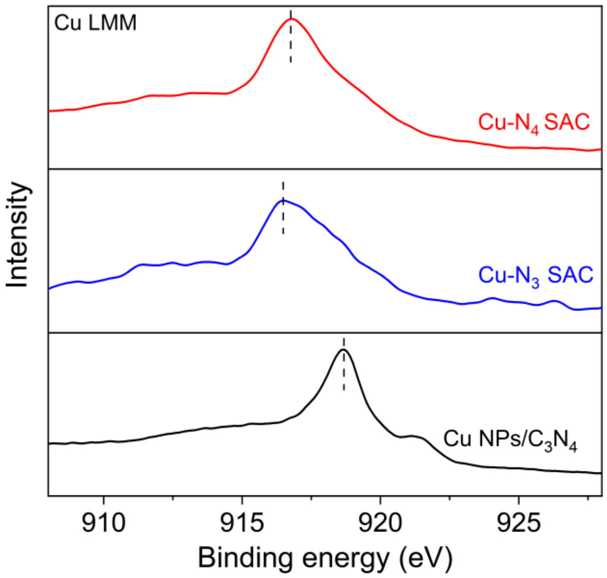

Supplementary Figure 9 | Structural analysis of various catalysts. Cu LMM of spectra of Cu–N_4 SAC, Cu–N_3 SAC and C_3N_4. Source data are provided as a Source Data file. Source data are provided as a Source Data file.

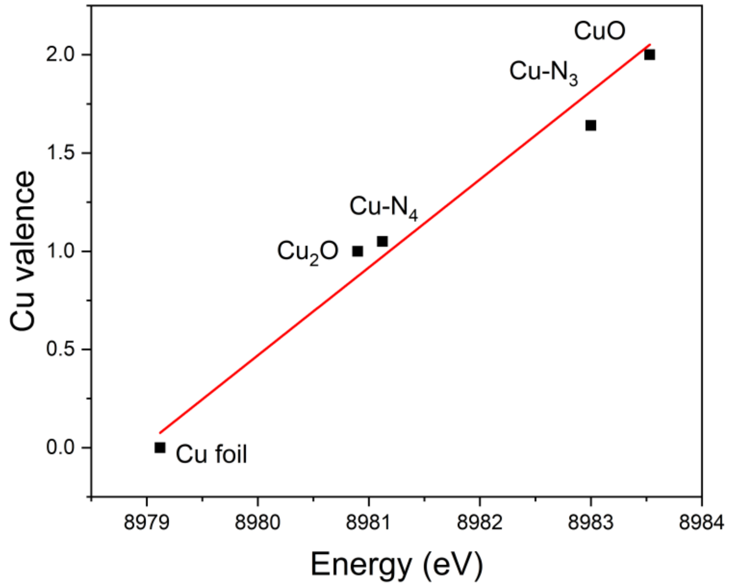

Supplementary Fig. 10 | The mean chemical valences of Cu species in Cu–N_3 and Cu–N_4 SACs. The values are calculated based XANES spectra of catalysts and references of Cu foil, Cu_2O and CuO. Source data are provided as a Source Data file.

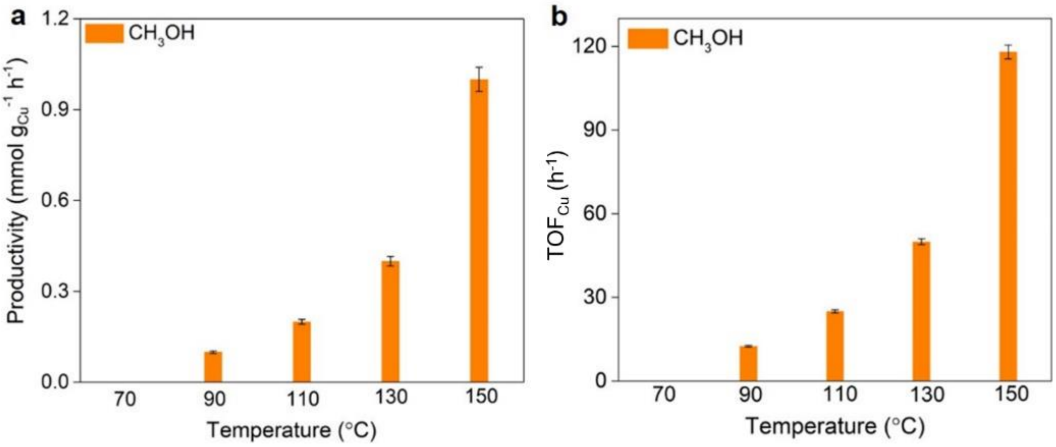

Supplementary Figure 11 | Catalytic performance of CO_2 hydrogenation over Cu NPs/C_3N_4. (**a**) CH_3OH productivities and (**b**) TOF values of Cu NPs/C_3N_4 at different temperatures. The reactor was pressurized with CO_2, H_2 and N_2 with a total pressure of 3.2 MPa (72:24:4 vol.%). All the data were collected for three times, and the error bars represent the standard deviation. Source data are provided as a Source Data file.

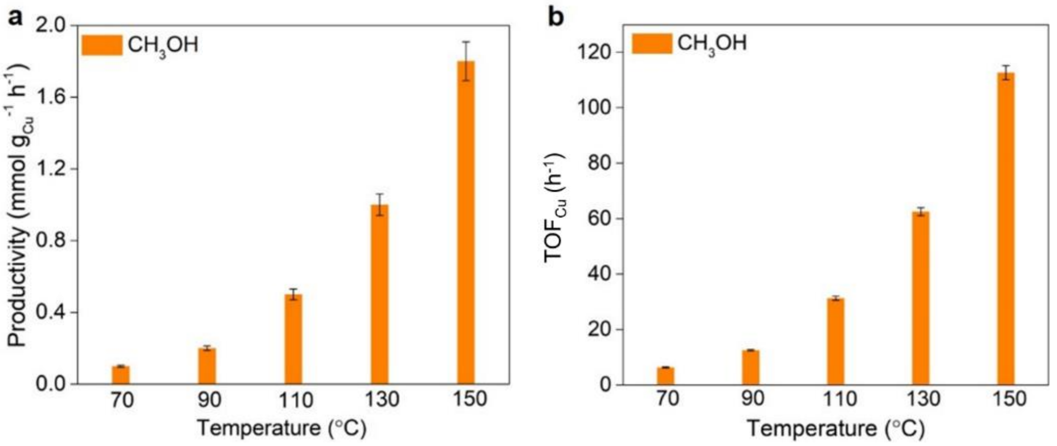

Supplementary Figure 12 | Catalytic performance of CO_2 hydrogenation over Cu-ZnO/Al_2O_3. (**a**) CH_3OH productivities and (**b**) TOF values of Cu-ZnO/Al_2O_3 at different temperatures. The reactor was pressurized with CO_2, H_2 and N_2 with a total pressure of 3.2 MPa (72:24:4 vol.%). All the data were collected for three times, and the error bars represent the standard deviation. Source data are provided as a Source Data file.

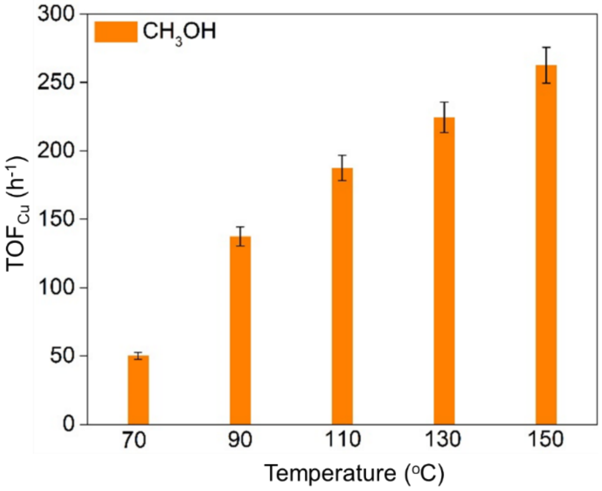

Supplementary Figure 13 | Catalytic performance of CO_2 hydrogenation over Cu–N_4 SAC. TOF values of CH_3OH for Cu–N_4 SAC in a temperature range of 70–150 ^{o}C. The reactor was pressurized with CO_2, H_2 and N_2 with a total pressure of 3.2 MPa (72:24:4 vol.%). All the data were collected for three times, and the error bars represent the standard deviation. Source data are provided as a Source Data file.

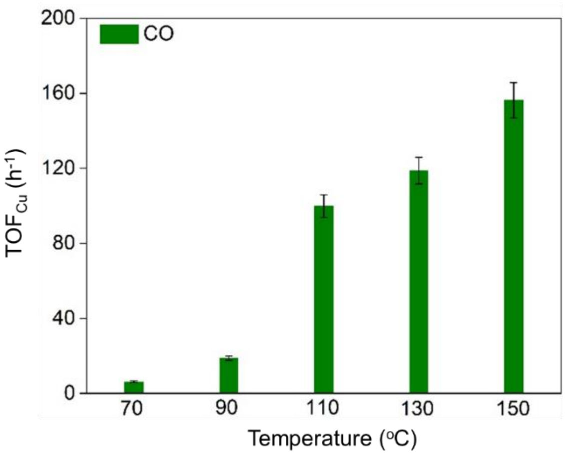

Supplementary Figure 14 | Catalytic performance of CO_2 hydrogenation over Cu–N_3 SAC. TOF values of CO for Cu–N_3 SAC in a temperature range of 70–150 °C. The reactor was pressurized with CO_2, H_2 and N_2 with a total pressure of 3.2 MPa (72:24:4 vol.%). All the data were collected for three times, and the error bars represent the standard deviation. Source data are provided as a Source Data file.

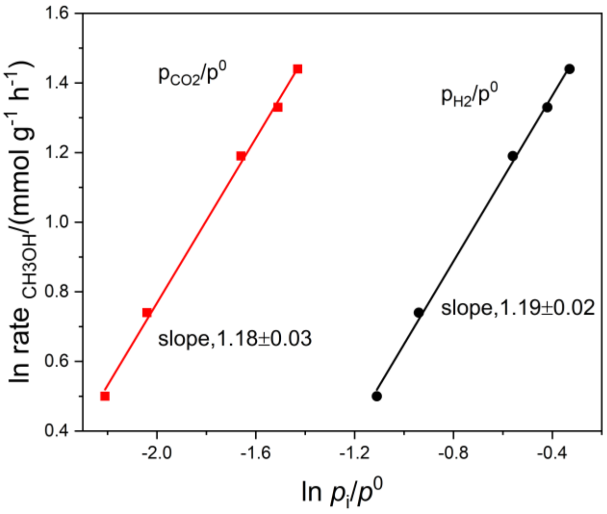

Supplementary Figure 15 | The reaction orders with respect to CO_2 and H_2 for Cu–N_4 SAC. Data were collected at 150 ºC and a total pressure of 3.2 MPa with a fixed CO_2/H_2 ratio of 3. Source data are provided as a Source Data file.

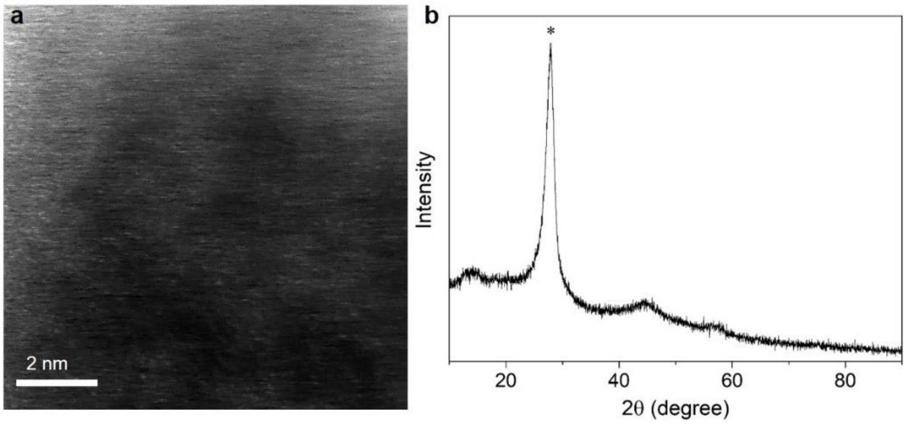

Supplementary Figure 16 | Characterizations of the spent Cu–N_4 SAC. (**a**) AC–TEM image (**b**) XRD pattern of the spent Cu–N_4 SAC was collected after 5 consecutive cycles at 150 °C. "*" represents the characteristic peak of C_3N_4. Source data are provided as a Source Data file.

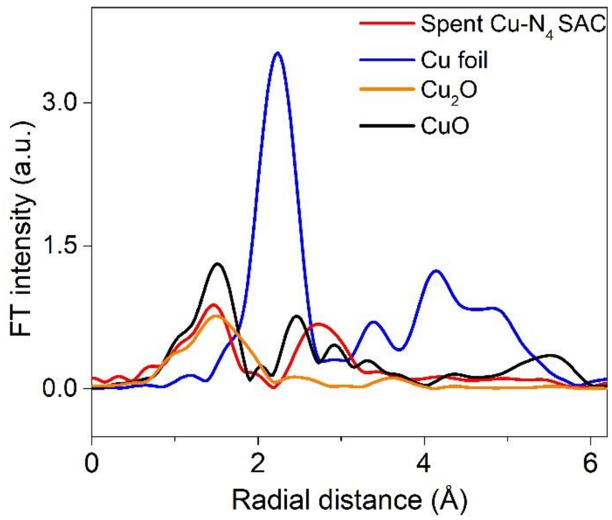

Supplementary Figure 17 | Characterizations of the spent Cu–N_4 SAC. EXAFS spectra were collected at Cu *K*-edge. Source data are provided as a Source Data file.

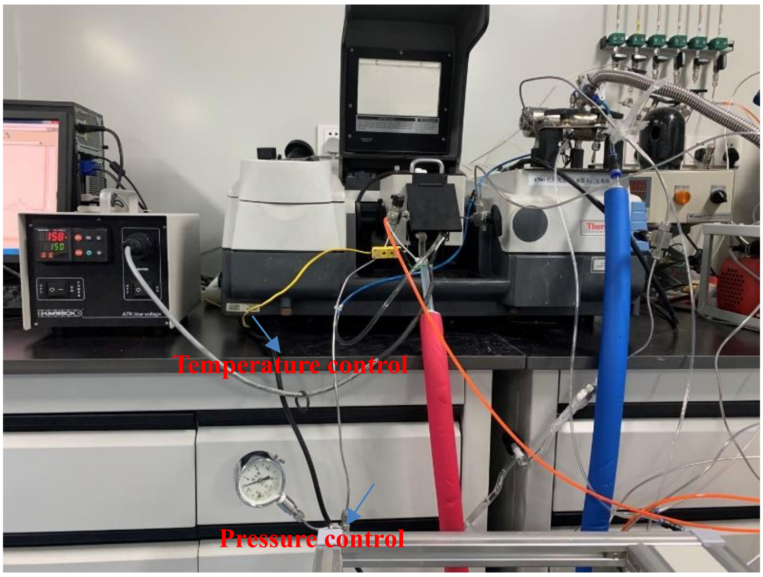

Supplementary Figure 18 | Photograph of in-situ infrared spectrometer. Note that the temperature and pressure can be regulated during test.

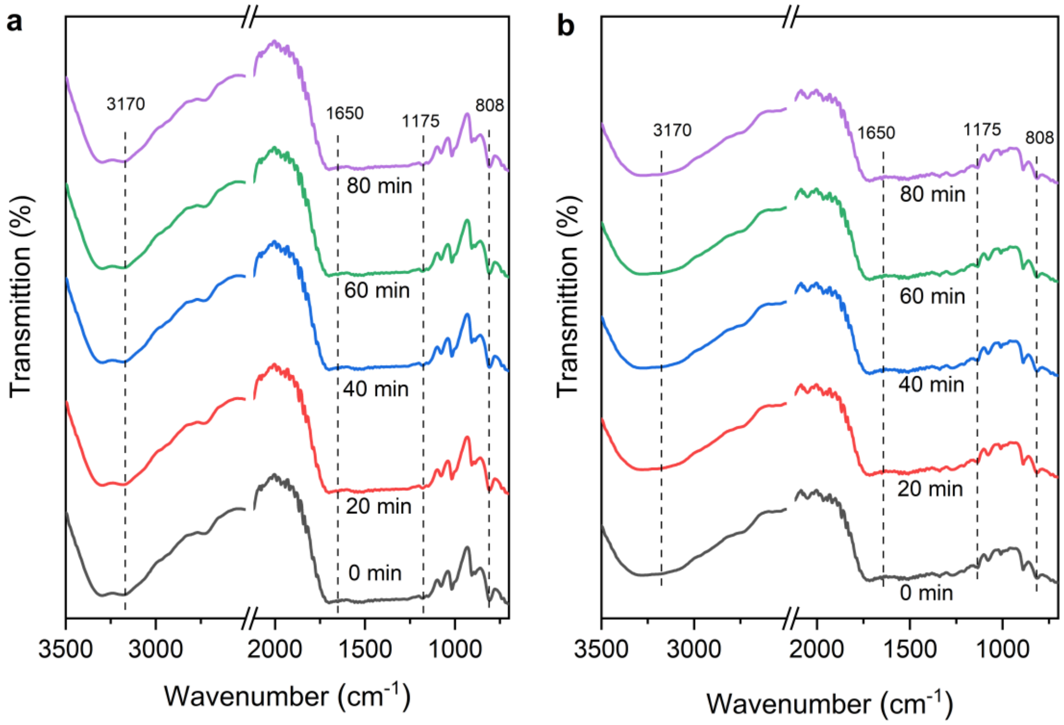

Supplementary Figure 19 | In situ DRIFTS spectra of SACs for CO_2 hydrogenation. In situ DRIFTS spectra of (a) Cu–N_4 and (b) Cu–N_3 SACs. The collected DRIFT spectra after exposing samples to the mixture of H_2, CO_2 and N_2 (72:24:4 vol.%) at 3 MPa and 150 °C. Source data are provided as a Source Data file.

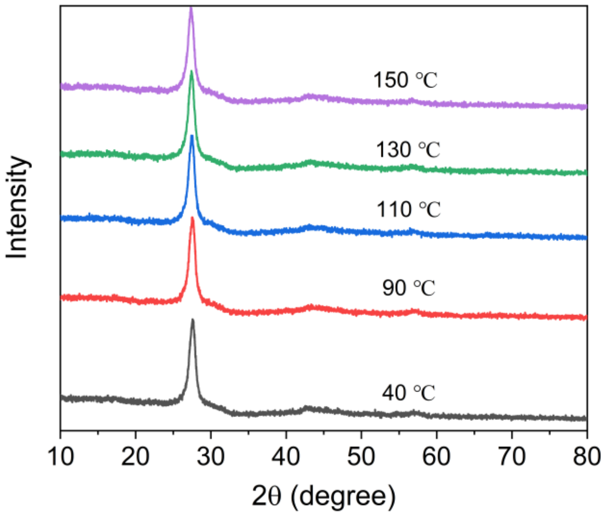

Supplementary Figure 20 | In situ XRD pattern of Cu–N_4 SAC. The experiment conducted at the mixture of H_2, CO_2 and N_2 (72:24:4 vol.%) at 0.03 MPa and with different temperature. Source data are provided as a Source Data file.

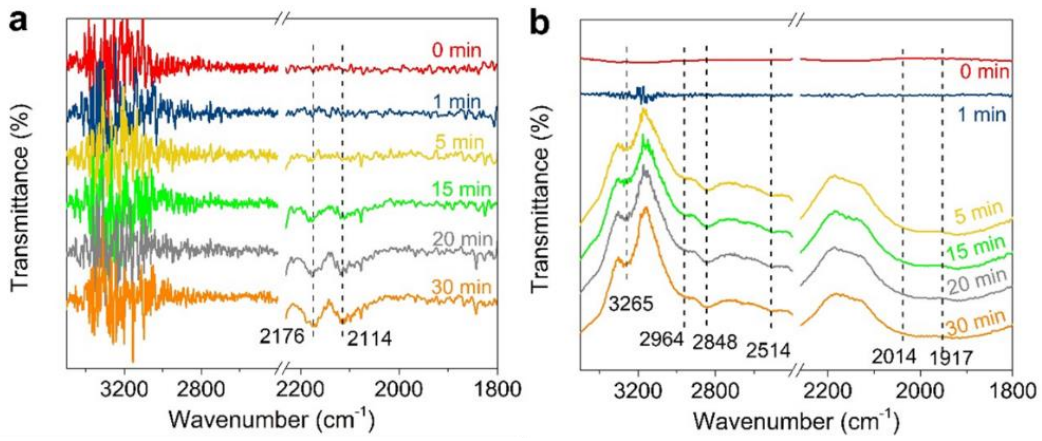

Supplementary Figure 21 | *In situ* DRIFTS spectra of SACs for CO_2 hydrogenation. *In situ* DRIFTS spectra of (**a**) Cu–N_4 and (**b**) Cu–N_3 SACs. The collected DRIFT spectra after exposing samples to the mixture of H_2, CO_2 and N_2 (72:24:4 vol.%) at atmospheric pressure and 150 °C. Source data are provided as a Source Data file.

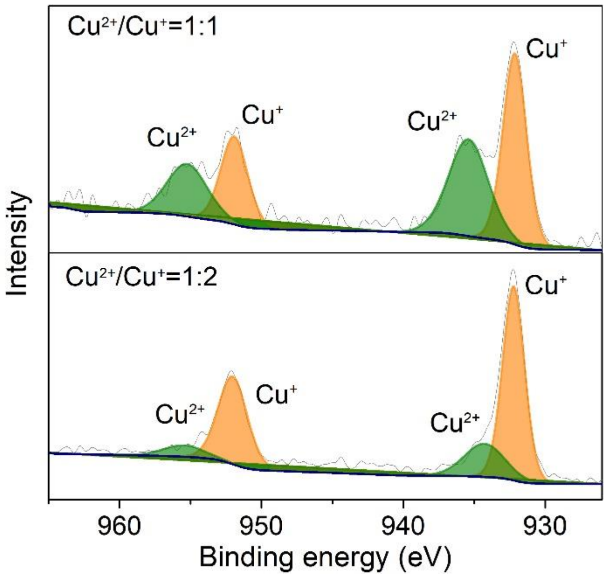

Supplementary Figure 22 | Cu 2p XPS spectra of catalysts with different ratio of Cu^{2+}:Cu^{+}. The variations of Cu^{2+}:Cu^{+} ratios were achieved by treating Cu–N_3 SAC at 500 °C in H_2 for different times (15 min for Cu^{2+}/Cu^{+} = 1:1 and 30 min for Cu^{2+}/Cu^{+} = 1:2). Source data are provided as a Source Data file.

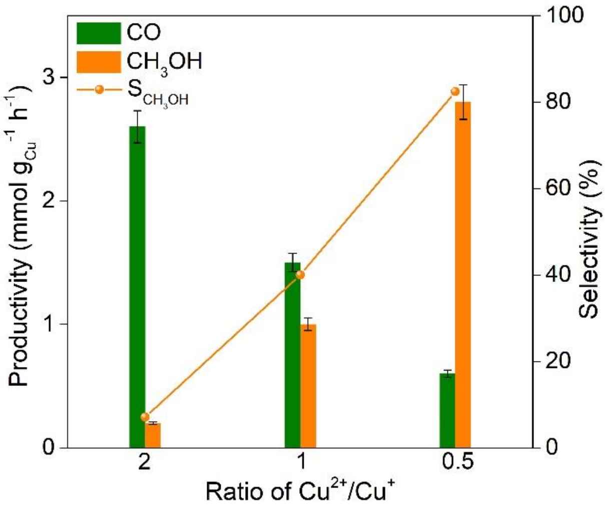

Supplementary Figure 23 | CO_2 hydrogenation performance over Cu-based SACs with different Cu^{2+}:Cu^{+} ratios. The reactor was pressurized with CO_2, H_2 and N_2 with a total pressure of 3.2 MPa (72:24:4 vol.%). All the data were collected for three times, and the error bars represent the standard deviation. Source data are provided as a Source Data file.

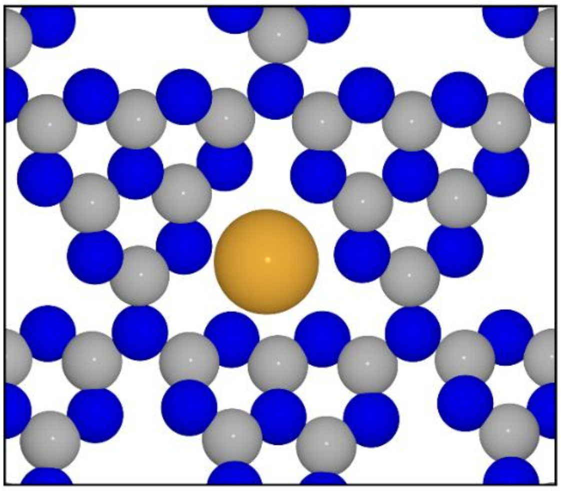

Supplementary Figure 24 | Atomic configuration of Cu–N_4 SAC. Cu single atom locates at the hole site of C_3N_4. Yellow: Cu atom, blue: N atoms, and grey: C atoms.

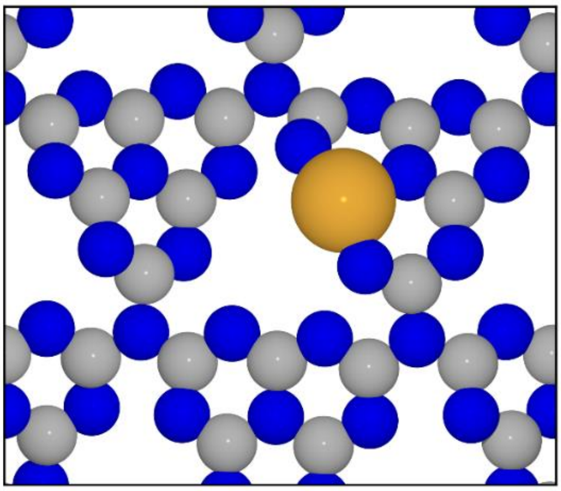

Supplementary Figure 25 | Atomic configuration of Cu–N_3 SAC. Cu atom replaces one of C atoms in C_3N_4. Yellow: Cu atom, blue: N atoms, and grey: C atoms.

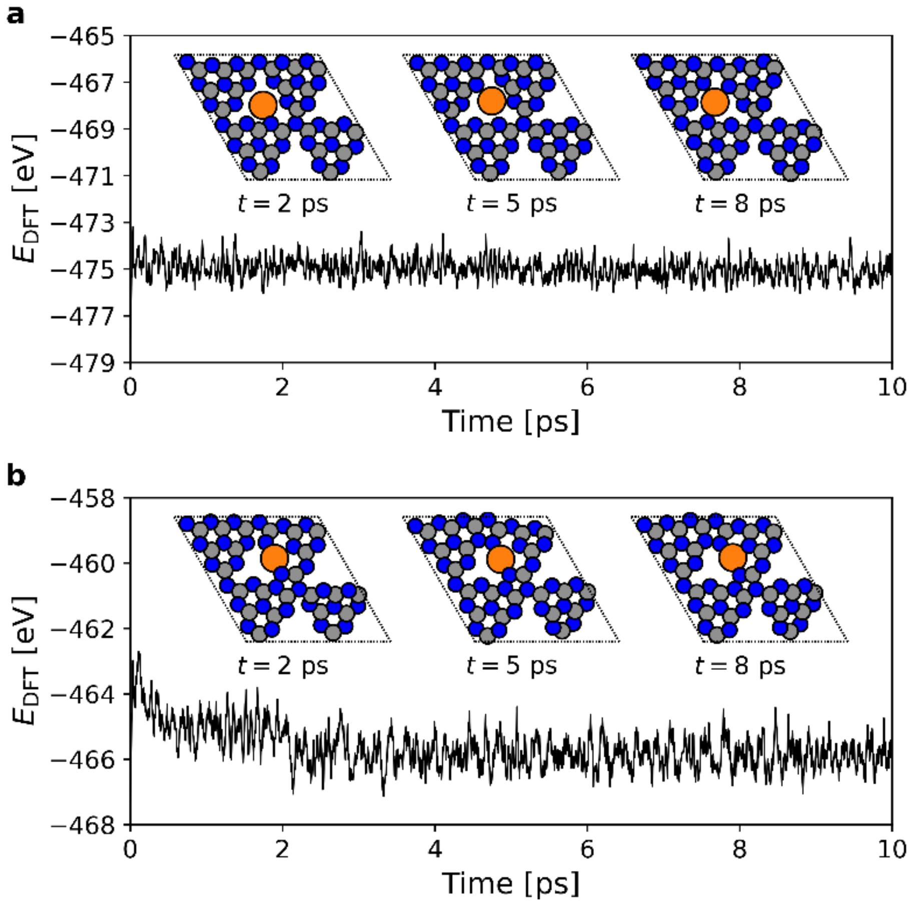

Supplementary Figure 26 | The DFT total energies of (a) Cu–N_4 and (b) Cu–N_3 structures vs. total simulation time at 450 K. The inset shows the structures at 2ps, 5 ps and 8 ps.

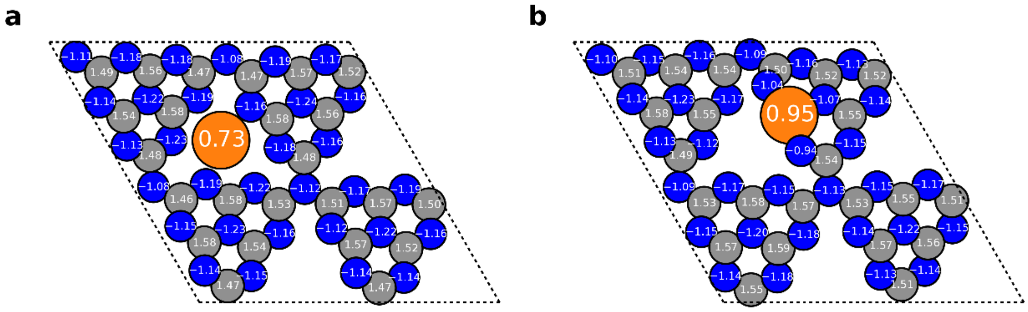

Supplementary Figure 27 | Bader charge analysis for (a) Cu–N_4 and (b) Cu–N_3 SACs. Yellow: Cu atom, blue: N atoms, and grey: C atoms.

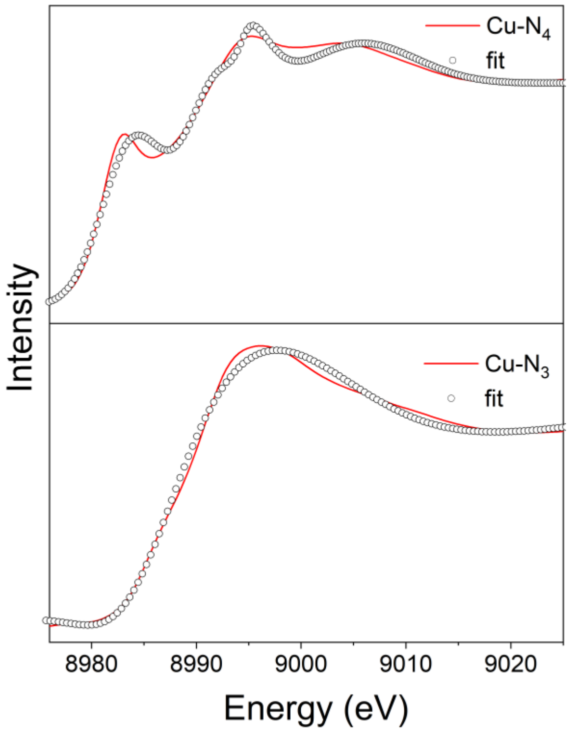

Supplementary Figure 28 | Simulated XANES spectra based on the selected models for DFT calculations. The structures of Cu–N_4 and Cu–N_3 are given in Supplementary Figures 24 and 25, respectively. Source data are provided as a Source Data file.

Supplementary Table 1 | Physicochemical properties of various catalysts.

Entry	Catalyst	Cu wt.%[a]
1	Cu–N_4	12.1
2	Cu–N_3	10.8
3	Cu NPs/C_3N_4	12.2

[a]: The compositions were determined by ICP-AES measurement.

Supplementary Table 2 | EXAFS fitting of fresh SACs.

Catalyst	Path	N	R (Å)	σ^2 (Å^2)	ΔE_0 (eV)	R factor
Cu–N_3 SAC	Cu–N	3.1 ± 0.2	1.870	0.0068 ± 0.0006	0.642±4.273	0.008
Cu–N_4 SAC	Cu–N	3.7 ± 0.4	1.896	0.0075 ± 0.0017	2.1 ± 1.3	0.008

Supplementary Table 3 | Comparison between Cu-C_3N_4 SACs and other reported catalysts for CO_2 hydrogenation

Catalyst	T (°C)	TOF (h^{-1})	Selectivity	Ref.
Cu-N_4 SAC	150	262.5	S_{CH3OH} = 95.5%	This work
Cu-N_3 SAC	150	156.3	S_{CO} = 94.3%	This work
Cu/Mo_2C	135	1.7	S_{CH3OH} = 93%	*J. Catal.* **2016**, *343*, 147
Pt/Co_3O_4	140	—	S_{C2+OH} = 82.5%	*Angew. Chem. Int. Ed.* **2016**, *55*, 737
$Rh_{75}W_{25}$	150	592	S_{CH3OH} = 97%	*Nano Lett.* **2017**, *17*, 788
Pt_4Co NWs/C	210	1418	—	*Small* **2017**, *13*, 1604311
Pt(3)/MoO_x(30)/TiO_2	150	—	S_{CH3OH} = 94.3%	*ACS Catal.* **2019**, *9*, 8187
Pt_1@MIL	150	117	S_{CH3OH} = 90.3%	*Nat. Commun*. **2019**, *10*, 1885
UiO-67-Pt	170-190	—	S_{CH3OH} = 42%	*J. Am. Chem. Soc.* **2020**, *142*, 17105
FL-MoS2	180	—	S_{CH3OH}=94.3%	*Nat. Catal.* **2021**, *4*, 242

Supplementary Table 4 | Free energy corrections (*T* = 423 K, *p* = 101325 Pa) for gas-phase species (in eV).

Species	E_{DFT}	E_{ZPE}	TS	Correction	G
H_2O	−14.220	0.568	0.881	0.000	−14.532
H_2	−6.771	0.269	0.617	0.000	−7.119
CO_2	−22.957	0.307	1.002	0.111	−23.541
CO	−14.776	0.132	0.912	−0.343	−15.900
CH_3OH	−30.226	1.354	1.127	0.000	−29.998

Supplementary Table 5 | Analysis of reaction enthalpies (in eV) of gas-phase thermochemical reactions. ΔH_{ref} values were taken from NIST, ΔH_{unc} values were calculated from PBE functional, ΔH_{cor} values were the correction values for reaction enthalpies.

Stoichiometry	ΔH_{ref}	ΔH_{unc}	$Error_{unc}$	Correction	ΔH_{cor}	$Error_{cor}$
$CO_2 + H_2 \rightarrow CO + H_2O$	0.427	0.861	−0.434	−0.454	0.407	0.019
$CO_2 + 4H_2 \rightarrow 2H_2O + CH_4$	−1.707	−1.649	−0.058	−0.111	−1.759	0.052
$3H_2 + CO \rightarrow H_2O + CH_4$	−2.134	−2.510	0.376	0.343	−2.167	0.033
$CO_2 + H_2 \rightarrow HCOOH$	0.152	0.075	0.077	0.000	0.075	0.077
$CO + H_2O \rightarrow HCOOH$	−0.274	−0.786	0.511	0.454	−0.332	0.058
$CO_2 + 3H_2 \rightarrow H_2O + CH_3OH$	−0.511	−0.513	0.002	−0.111	−0.624	0.113
$2H_2 + CO \rightarrow CH_3OH$	−0.938	−1.374	0.437	0.343	−1.031	0.094
$CO_2 + 3H_2 \rightarrow 2H_2O + 1/2C_2H_4$	−0.662	−0.572	−0.091	−0.111	−0.682	0.020
$2H_2 + CO \rightarrow H_2O + 1/2C_2H_4$	−1.089	−1.433	0.344	0.343	−1.090	0.001
$CO_2 + 7/2H_2 \rightarrow 2H_2O +1/2C_2H_6$	−1.369	−1.318	−0.051	−0.111	−1.428	0.059
$5/2H_2 + CO \rightarrow H_2O + 1/2C_2H_6$	−1.796	−2.179	0.383	0.343	−1.836	0.040
