# Supplementary material for: Coordination tailoring of Cu single sites on C3N4 realizes selective CO2 hydrogenation at low temperature
Source: Nat Commun. 2021 Oct 15;12:6022. doi: 10.1038/s41467-021-26316-6 (PMC8519910; doi:10.1038/s41467-021-26316-6)
Supplement: Supplementary file 2 — Reporting Summary [file 41467_2021_26316_MOESM2_ESM.pdf]

nature portfolio

Corresponding author(s): Yong Xu

Last updated by author(s): Sep 6, 2021

Reporting Summary

Nature Portfolio wishes to improve the reproducibility of the work that we publish. This form provides structure for consistency and transparency in reporting. For further information on Nature Portfolio policies, see our Editorial Policies and the Editorial Policy Checklist.

Statistics

For all statistical analyses, confirm that the following items are present in the figure legend, table legend, main text, or Methods section.

n/a	Confirmed	
☒	☐	The exact sample size (*n*) for each experimental group/condition, given as a discrete number and unit of measurement
☒	☐	A statement on whether measurements were taken from distinct samples or whether the same sample was measured repeatedly
☒	☐	The statistical test(s) used AND whether they are one- or two-sided<br>*Only common tests should be described solely by name; describe more complex techniques in the Methods section.*
☒	☐	A description of all covariates tested
☒	☐	A description of any assumptions or corrections, such as tests of normality and adjustment for multiple comparisons
☒	☐	A full description of the statistical parameters including central tendency (e.g. means) or other basic estimates (e.g. regression coefficient) AND variation (e.g. standard deviation) or associated estimates of uncertainty (e.g. confidence intervals)
☒	☐	For null hypothesis testing, the test statistic (e.g. *F*, *t*, *r*) with confidence intervals, effect sizes, degrees of freedom and *P* value noted<br>*Give P values as exact values whenever suitable.*
☒	☐	For Bayesian analysis, information on the choice of priors and Markov chain Monte Carlo settings
☒	☐	For hierarchical and complex designs, identification of the appropriate level for tests and full reporting of outcomes
☒	☐	Estimates of effect sizes (e.g. Cohen's *d*, Pearson's *r*), indicating how they were calculated

Our web collection on statistics for biologists contains articles on many of the points above.

Software and code

Policy information about availability of computer code

Data collection	DFT calculations were performed by using the Perdew-Burke-Ernzerhof (PBE) functional within the generalized gradient approximation (GGA) implemented in Vienna Ab Initio Simulation Package (VASP).
Data analysis	In DFT calculations, the projector-augmented wave (PAW) method was applied to describe the electron-ion interactions, and the D3 Grimme's method was employed to correct van der Waals interaction. We used a plane-wave cutoff energy of 520eV and the Gaussian smearing with a width of 0.05 eV. Periodic boundary conditions were applied, and more than 15 Å of vacuum space was used to avoid the interaction of the adjacent images. A Γ-centered (3, 3, 1) k-point grid was adopted to sample the Brillouin zone of a 2×2 buckled C3N4 supercell with the lattice constant of 6.94 Å. All the structures were fully relaxed until the force components were less than 0.02 eV·Å–1.

For manuscripts utilizing custom algorithms or software that are central to the research but not yet described in published literature, software must be made available to editors and reviewers. We strongly encourage code deposition in a community repository (e.g. GitHub). See the Nature Portfolio guidelines for submitting code & software for further information.

Data

Policy information about availability of data

All manuscripts must include a data availability statement. This statement should provide the following information, where applicable:

- Accession codes, unique identifiers, or web links for publicly available datasets
- A description of any restrictions on data availability
- For clinical datasets or third party data, please ensure that the statement adheres to our policy

The authors declare data supporting the findings of this study are available within the paper and its Supplementary Information. All data are available from the authors on reasonable request.

Field-specific reporting

Please select the one below that is the best fit for your research. If you are not sure, read the appropriate sections before making your selection.

☐ Life sciences ☐ Behavioural & social sciences ☒ Ecological, evolutionary & environmental sciences

For a reference copy of the document with all sections, see nature.com/documents/nr-reporting-summary-flat.pdf

Ecological, evolutionary & environmental sciences study design

All studies must disclose on these points even when the disclosure is negative.

Study description	We demonstrate here that the C3N4 supported Cu single atom catalysts (SACs) with tailored coordination environment, namely, Cu–N4 and Cu–N3 SACs, can be applied as highly selective and active CO2 hydrogenation catalysts at low temperature, in which the modulation of the coordination structure of Cu atoms in SAC is readily realized by altering the treatment parameters. Significantly, the resulting C3N4 supported Cu SACs show highly coordination environment dependent selectivity towards CO2 hydrogenation, where the Cu–N4 SAC exhibits a CH3OH selectivity of 95.5%, while Cu–N3 SAC displays a CO selectivity of 94.3% for CO2 hydrogenation.
Research sample	C3N4 supported Cu single atom catalyst
Sampling strategy	The products were injected into gas chromatography in the presence of carrier gas.
Data collection	The gaseous mixture was analyzed using a gas chromatograph (Shiweipx GC-7806) equipped with a GDX-502 column connected to a thermal conductivity detector. The liquid mixture was collected by centrifugation at 12000 rpm for 3 min. 10 μL isopropanol was introduced into 1 mL reaction mixture as an internal standard. The liquid mixture was analysed using a gas chromatograph (Persee G5) equipped with a KB-5 column connected to a flame ionization detector. The tests were repeated three times for each catalyst.
Timing and spatial scale	NA
Data exclusions	No data were excluded.
Reproducibility	The data during catalytic tests were collected for three times.
Randomization	NA
Blinding	All the data were analyzed by gas chromatography .

Did the study involve field work? ☐ Yes ☒ No

Reporting for specific materials, systems and methods

We require information from authors about some types of materials, experimental systems and methods used in many studies. Here, indicate whether each material, system or method listed is relevant to your study. If you are not sure if a list item applies to your research, read the appropriate section before selecting a response.

Materials & experimental systems

n/a	Involved in the study	
☒	☐	Antibodies
☒	☐	Eukaryotic cell lines
☒	☐	Palaeontology and archaeology
☒	☐	Animals and other organisms
☒	☐	Human research participants
☒	☐	Clinical data
☒	☐	Dual use research of concern

Methods

n/a	Involved in the study	
☒	☐	ChIP-seq
☒	☐	Flow cytometry
☒	☐	MRI-based neuroimaging
